# Exploring the Reasons for Decrease in Binding Affinity of HIV-2 Against HIV-1 Protease Complex Using Interaction Entropy Under Polarized Force Field

**DOI:** 10.3389/fchem.2018.00380

**Published:** 2018-08-24

**Authors:** Yalong Cong, Yuchen Li, Kun Jin, Susu Zhong, John Z. H. Zhang, Hao Li, Lili Duan

**Affiliations:** ^1^School of Physics and Electronics, Shandong Normal University, Jinan, China; ^2^Shanghai Engineering Research Center of Molecular Therapeutics and New Drug Development, School of Chemistry and Molecular Engineering, East China Normal University, Shanghai, China; ^3^NYU-ECNU Center for Computational Chemistry at NYU Shanghai, Shanghai, China; ^4^Department of Chemistry, New York University, New York, NY, United States; ^5^Department of Science and Technology, Shandong Normal University, Jinan, China

**Keywords:** molecular dynamics simulations, binding free energy calculation, polarized force field, HIV protease, interaction entropy

## Abstract

In this study, the differences of binding patterns between two type HIV (HIV-1 and HIV-2) protease and two inhibitors (darunavir and amprenavir) are analyzed and compared using the newly developed interaction entropy (IE) method for the entropy change calculation combined with the polarized force field. The functional role of protonation states in the two HIV-2 complexes is investigated and our study finds that the protonated OD1 atom of Asp25′ in B chain is the optimal choice. Those calculated binding free energies obtained from the polarized force field combined with IE method are significantly consistent with the experimental observed. The bridging water W301 is favorable to the binding of HIV-1 complexes; however, it is unfavorable to the HIV-2 complexes in current study. The volume of pocket, B-factor of Cα atoms and the distance of flap tip in HIV-2 complexes are smaller than that of HIV-1 consistently. These changes may cause localized rearrangement of residues lining their surface and finally result in the different binding mode for the two types HIV. Predicated hot-spot residues (Ala28/Ala28′, Ile50/Ile50′, and Ile84/Ile84′) are nearly same in the four systems. However, the contribution to the free energy of Asp30 residue is more favorable in HIV-1 system than in HIV-2 system. Current study, to some extent, reveals the origin for the decrease in binding affinity of inhibitors against HIV-2 compared with HIV-1 and will provides theoretical guidance for future design of potent dual inhibitors targeting two type HIV protease.

## Introduction

Acquired immunodeficiency syndrome (AIDS) has been a major global health challenge due to its serious threat to human life. According to data of The Joint United Nations Programme on HIV/AIDS (UNAIDS), around 36.7 million people within the worldwide are infected with HIV in recent years. Currently, there are two major etiological causative agents of AIDS: human immunodeficiency virus type-1 (HIV-1) and type-2 (HIV-2). As the most common type, HIV-1 which is divided into four groups (M, N, O, and P) and several subtypes (Tie et al., 2012), is observed around the global. Another type, HIV-2, is firstly identified in West Africa (Guyader et al., 1987; Menéndez-Arias and Tözsér, 2008; Peterson et al., 2011), but now it is found which is gradually spreading to other parts of the world (Soriano et al., 2000; Barin et al., 2007; Kovalevsky et al., 2008).

HIV-1 protease (PR1) (Navia et al., 1989) is extremely effective as a drug target for AIDS treatment (Debouck, 1992). It plays an essential role in the life cycle of HIV by cleaving the Gag and Gag-pol non-functional polypeptides into mature and infectious HIV viral particles (Wlodawer and Vondrasek, 1998; Louis et al., 2000). PR1 inhibitors can combine preemptively the active-site cavity of PR1, and at the same time, it can prevent the hydrolysis of the viral Gag and Gal-Pol polypeptides, which results in immature and non-infectious viral particles. However, due to the lack of specifically targeted inhibition for HIV-2 protease (PR2), the study for those patients infected by HIV-2 faces significant challenges. PR1 and PR2 share ~50–70% sequence identity (Gustchina and Weber, 1991; Tözsér et al., 1991) and they have very similar tertiary structure, while PR1 inhibitors show lower efficiency and weaker inhibition for PR2 (Brower et al., 2008; Peterson et al., 2011). Therefore, the design of new inhibitor specially targeting PR2 is urgent for those treatment of patients infected by HIV-2.

**Figure 1:**
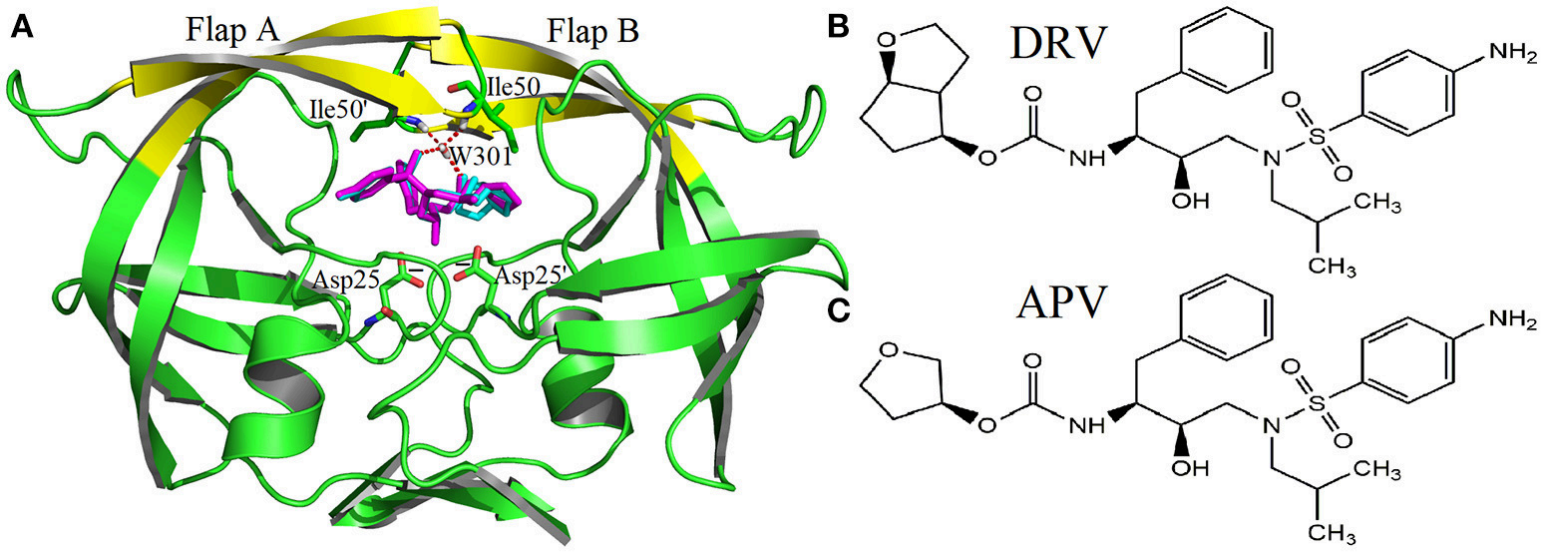
The native structures of molecular: **(A)** complexes of PR2 and two inhibitors (DRV is cyan, APV is magenta); **(B)** Inhibitor DRV; **(C)** Inhibitor APV.

The family of HIV protease (PR) is a kind of homodimer enzyme, which consists of two identical 99 amino acid monomers. The flap regions (flap A: residues 43–58 of A chain, flap B: 43'-58' of B chain) form two β hairpin structures at the active site, respectively. They control the substrate to enter or leave the substrate-binding cavity by closing or opening pocket of cavity (Miller et al., 1989; Hornak et al., 2006b). Furthermore, the bridging water W301 plays a significant role in opening and closing of the flap as well as the binding for HIV and inhibitor (Lu et al., 2006; Barillari et al., 2007; Duan et al., 2007). W301 forms four hydrogen bonds at the active site together, two with flap tip (Ile50 and Ile50′) and two with inhibitor (shown in Figure 1). In addition, PR has five alternative protonation states: unprotonation (unpro), mono protonation of Asp25 of A chain on OD1 (25OD1), mono protonation of Asp25 on OD2 (25OD2), mono protonation of Asp25′ of B chain on OD1 (25′OD1), and mono protonation of Asp25′ on OD2 (25′OD2). For PR1, the mono protonation state is generally assigned to OD2 of Asp25′ (Hou and Yu, 2007; Chen et al., 2014; Duan et al., 2015); however, the protonation state of PR2 is unknown up to now.

Although many theorists and experimenters have studied the binding mechanism of PR1 and inhibitors, the research about PR2 is still deficient. Clavel et al. analyzed the nucleotide sequence of the human retrovirus associated with PR2 and found that their biological properties are conserved in spite of limited sequence homology in PR1 and PR2 system (Guyader et al., 1987). Weber et al. compared sequences and structures of PR1 and PR2 and found that the origin of difference in affinity was partly attributable to the change of Val32 in PR1 to Ile32 in PR2 (Gustchina and Weber, 1991). Freire et al. performed kinetic inhibition assays to measure the inhibition constants (Ki) of the HIV-1 protease inhibitors against the HIV-2 protease and found that inhibitors show Ki ratios ranging between 2 and 80 for HIV-2 and HIV-1 proteases, respectively (Brower et al., 2008). Recently, Regad et al. compared the binding pockets of PR1 and PR2 and found that pockets of bound PR2 were more hydrophobic with more oxygen atoms and fewer nitrogen atoms than pockets of PR1 (Triki et al., 2018). In this paper, our work focuses on the investigation for the differences of binding patterns between two PRs and inhibitors, besides discussing the origin of the decrease in affinity of two inhibitors against HIV-2 protease compared with HIV-1 protease by molecular dynamics (MD) simulation and the binding free energy calculation.

As we know, the accuracy of the dynamics results obtained from MD simulation mainly depends on the accuracy of the force field adopted. Current standard force fields, such as AMBER, GROMACS, CHARMM and so on, lack the electronic polarization effect and this leads to inaccurate and unreliable results. Recent years, some polarizable force fields (Halgren and Damm, 2001), such as AMOEBA (Ponder et al., 2010) in AMBER package, have been gradually developed, however, the practical application is more complicated than non-polarizable force field. Currently, several models including polarization effects, such as the fluctuating charge model (Banks et al., 1998), induced multipole (Ren and Ponder, 2003), Drude oscillator (Lamoureux et al., 2003), and quantum mechanical treatment (Gordon et al., 2013), have been proposes. The uncertainty of the accuracy and validity of the basic theoretical model used to derive polarizable force field still exists (Warshel et al., 2007). In our study, the polarized protein-specific charge (PPC) force field (Ji et al., 2008) based on quantum mechanical calculation is employed. It can provide more accurate electrostatic interaction for PR and inhibitor than traditional non-polarizable force field. Extensive studies have also demonstrated that the electronic polarization effect plays an essential role in MD simulations (Ji and Zhang, 2008; Duan et al., 2010, 2016a; Ji and Mei, 2014).

In the binding free energy calculation, it is widely believed that free energy perturbation (FEP) (Bash et al., 1987; Rao et al., 1987) and thermodynamic integration (TI) (Straatsma and Berendsen, 1988; Åqvist et al., 1994) are the most accurate and rigorous methods among all methods. However, the low computational efficiency and the lack of the ability to calculate absolute free energy are the common shortcomings in the two methods. In contrast, as another effective method for free energy calculation, the molecular mechanics Poisson-Boltzmann surface area (MM/PBSA) (Srinivasan et al., 1998; Wang and Kollman, 2000; Wang J. et al., 2001; Wang W. et al., 2001; Xu et al., 2013; Sun et al., 2014; Genheden and Ryde, 2015; Chen et al., 2016) has become a very popular method duo to its full functioning and efficiency in the absolute free energy calculation. However, the entropy change calculation in MM/PBSA method is suffering the developmental bottleneck. The standard normal mode (Nmode) (Nguyen and Case, 1985) in the MM/PBSA is time-consuming and approximate for the entropic calculation. The method considers the internal motions of protein as superposition of vibrations with different frequencies (Xu et al., 2011), and then calculates the vibrational entropy. Besides, the calculation of Hessian matrix of the energy (second derivative of energy) is extremely costly in multiple degrees of freedom (Yan et al., 2017). Ray Luo, the developer of MM/PBSA, finds that the normal mode approximation does not benefit too much to the quality of the MM/PBSA calculations (Wang et al., 2016). So, it is promising to further improve the accuracy of MM/PBSA methods by improving the accuracy of the calculation of entropy change using a highly efficient and reliable method. In this paper, a novel and effective method, namely interaction entropy (IE) (Duan et al., 2016b, 2017), is used to calculate the entropy change. The method is more theoretically rigorous and time-saving than traditional Nmode method (Cong et al., 2017; Yan et al., 2017; Liu et al., 2018; Qiu et al., 2018; Song et al., 2018).

In addition, as the main tool to search for hot-spots residues, the residue decomposition of binding free energy in MM/GBSA method has always been used to explore the binding mechanism of protein and ligand. However, it is difficult to decompose entropy change into each residue using traditional Nmode method. Therefore, the effect of entropic contribution on the calculation for predicting hot-spots residues is generally ignored. Fortunately, the IE method is able to calculate straightforwardly the entropy change of each residue. The combination of MM/GBSA and IE will further provide detailed and reliable information for predicting hot spots and understanding the interaction mechanism between protein and ligand.

## Method

### Polarized protein-specific charge

PPC force field based on quantum mechanical calculation can provide accurate partial atomic charges of proteins to represent electrostatic polarization effect. With the help of molecular fragmentation using conjugated caps scheme (MFCC) method (Zhang and Zhang, 2003), we firstly cleave the protein into fragments at the peptide bond and add a pair of conjugate caps to achieve the electronic structure of protein by fully quantum mechanical (QM). Next the restrained electrostatic potentials (RESP) (Bayly et al., 1993) program is employed to fit atomic charges. Then the Poisson-Boltzmann (PB) Equation (Tannor et al., 1994) is used to calculate electrostatic solvation energy and generate discrete induced charges on cavity surface. The newly obtained charges of other residues and surface charges mimicking the solvent effect are regarded as background charges in the next cycle of QM calculation for each fragment. Finally, the solute and solvent polarize each other until solvation energy and induced charges converge.

### MM/PBSA

As one of the most widely used methods, MM/PBSA has always played a significant role on calculation of binding free energy. In the MM/PBSA approach, the binding free energy Δ*G*_*bind*_ is calculated as follows:

(1) $$\begin{array}{r} \Delta G_{bind}=\Delta H-T\Delta S=\langle E_{pl}^{int}\rangle +\Delta G_{sol}-T\Delta S \end{array}$$

where the Δ*H* represents enthalpy change, and the $\langle E_{pl}^{int}\rangle$ represents the ensemble averaged protein-ligand interaction including electrostatic interaction and van der Waals (vdW) interaction. Δ*G*_*sol*_ and −*TΔS* represent the solvation free energy and contribution of entropy change, respectively. Among them, the Δ*G*_*sol*_ can be divided into two parts:

(2) $$\begin{array}{r} \Delta G_{sol}=\Delta G_{pb}+\Delta G_{np} \end{array}$$

where Δ*G*_*pb*_ and Δ*G*_*np*_ represent the polar and non-polar solvation free energy, respectively. The Δ*G*_*pb*_ can be calculated through the PB equation. In our calculation, the exterior and interior dielectric constants are set to 80 and 1, respectively. In the meantime, the Δ*G*_*np*_ can be calculated through the following equation:

(3) $$\begin{array}{r} \Delta G_{np}=\gamma \times SASA+\beta \end{array}$$

where *SASA* represents solvent-accessible surface area, and it can be calculated by MSMS program (Sanner et al., 1996). The numerical values of γ and β are the standard values of 0.00542 kcal/(mol · Å^2^) and 0.92 kcal/mol, respectively.

In our works, the calculation of enthalpy change is performed based on 100 snapshots from MD trajectories. Considering that the calculation of entropy change is extremely time-consuming, we only extract averagely 20 snapshots from the above 100 snapshots to finish the calculation of entropy change by Nmode method.

### Interaction entropy

In addition to Nmode, a new more rigorous and concise method, interaction entropy (IE), is employed to calculate entropy change. It can be defined as:

(4) $$\begin{array}{r} -T\Delta S=KTln\langle e^{\beta \Delta E_{pl}^{int}}\rangle \end{array}$$

where $\Delta E_{pl}^{int}$ represents the fluctuation of protein-ligand interaction energy ($E_{pl}^{int}$) around the average energy ($\langle E_{pl}^{int}\rangle$). It can be calculated as:

(5) $$\begin{array}{r} \Delta E_{pl}^{int}=E_{pl}^{int}-\langle E_{pl}^{int}\rangle \end{array}$$

the protein-ligand interaction energy ($E_{pl}^{int}$) consists of electrostatic interaction and vdW interactions. The efficiency of this approach lies in that the two averages $\langle E_{pl}^{int}\rangle$ and $\langle e^{\beta \Delta E_{pl}^{int}}\rangle$ can be calculated simply by the following equations:

(6) $$\begin{array}{r} \langle E_{pl}^{int}\rangle =\frac{1}{N}\overset{N}{\underset{i}{\sum }}E_{pl}^{int}\left(t_{i}\right) \end{array}$$

and

(7) $$\begin{array}{r} \langle e^{\beta \Delta E_{pl}^{int}}\rangle =\frac{1}{N}\overset{N}{\underset{i}{\sum }}e^{\beta \Delta E_{pl}^{int}\left(t_{i}\right)} \end{array}$$

where *β* is 1/*KT*.

In the IE method, the residue decomposition of entropy change (Wang et al., 2017; Yan et al., 2017) is performed by the following equations:

(8) $$\begin{array}{r} -T\Delta S_{rl}=KTln\langle e^{\beta \Delta E_{rl}^{int}}\rangle \end{array}$$

where $\Delta E_{rl}^{int}$ represents the fluctuation of residues-ligand interaction energy ($E_{rl}^{int}$) around the average energy ($\langle E_{rl}^{int}\rangle$).

### MD simulation

The initial structures of DRV-PR1, DRV-PR2, AVP-PR1, and AVP-PR2 is obtained from the Protein Databank (PDB entry: 4LL3, 3EBZ, 3EKV, and 3S45, respectively). All water molecules, but the bridging water molecule W301, are removed from initial structures. For PR1, the mono protonated state is assigned to OD2 of Asp25′; For PR2, the five alternative protonation states all are considered. The geometry of ligand is optimized at the HF/6-31G^**^ level using Gaussian03 program (Frisch et al., 2003). Single point energy is calculated at the B3LYP/cc-PVTZ level to generate electrostatic potentials (ESP) and the atomic charges are fitted using the restrained ESP (RESP) (Cornell et al., 1993) method. The parameters of the protein and ligands are generated based on AMBER12SB force field and the general AMBER force field (GAFF) (Hornak et al., 2006a), respectively. The TIP3P water box is chosen as the solvent environment with 10 Å buffer between the complex and extremity of the water box. Counterions are added to keep the system electrically neutral. Energy minimization is carried out by the steepest descent method followed by the conjugate gradient minimization to remove bad contacts between the solute and solvent water molecules. Firstly, solvent water molecules are optimized by holding the complex fixed with force constant of 500 *kcal*/(*mol* · Å^2^). Secondly, the entire system is energy minimizes without any restrictions.

Before running MD simulation, the whole system is heated from 0 to 300 k with 10 *kcal*/(*mol* · Å^2^) harmonic constraints on all solute atoms for 300 ps. SHAKE (Ryckaert et al., 1977) algorithm is used to constrain all chemical bonds involving hydrogen atoms. Finally, MD simulation is performed for 100 ns in the explicit water using AMBER16 (Case et al., 2016). In the first 80 ns, the time of writing to the coordinated file is set to 4 ps. In the last 20 ns, the time of writing to the coordinated file is set to10 fs to obtain enough conformational sampling. The MD simulations using PPC force field is the same with AMBER force field except that the standard charges from AMBER force field are simply replaced by the PPC method.

## Results and analyses

### Analysis of the stability

In order to evaluate the stability of the system during MD simulations, the root mean square deviation (RMSD) of the protein backbone atoms relative to the corresponding crystal structure is calculated. And these results are shown on Figures S1, S2 in the Supporting Information. Obviously, the values of RMSD are fluctuated between 0.5 and 1.5 Å, which indicates that simulation has reached equilibrium. Moreover, the distances between W301 and inhibitor are calculated and plotted on Figures S3, S4 in the Supporting Information. It can be clearly observed in the six systems for DRV-PR2(unpro), DRV-PR2(25OD1), DRV-PR2(25OD2), APV-PR1(25′OD2), APV-PR2(unpro), and APV-PR2(25OD1), the bridging water W301 leaves the binding pocket after around 50, 80, 70, 20, 60, and 20 ns under AMBER force field, respectively. And there is no other water molecule taking its place in the subsequent MD simulation. Therefore, the above RMSD obtained from AMBER force field is calculated before the bridging water is far away from the binding pocket. In contrast, W301 tightly binds to its original position under PPC simulation using the fluctuation of hydrogen bond length for about 3.5 Å.

**Figure 2:**
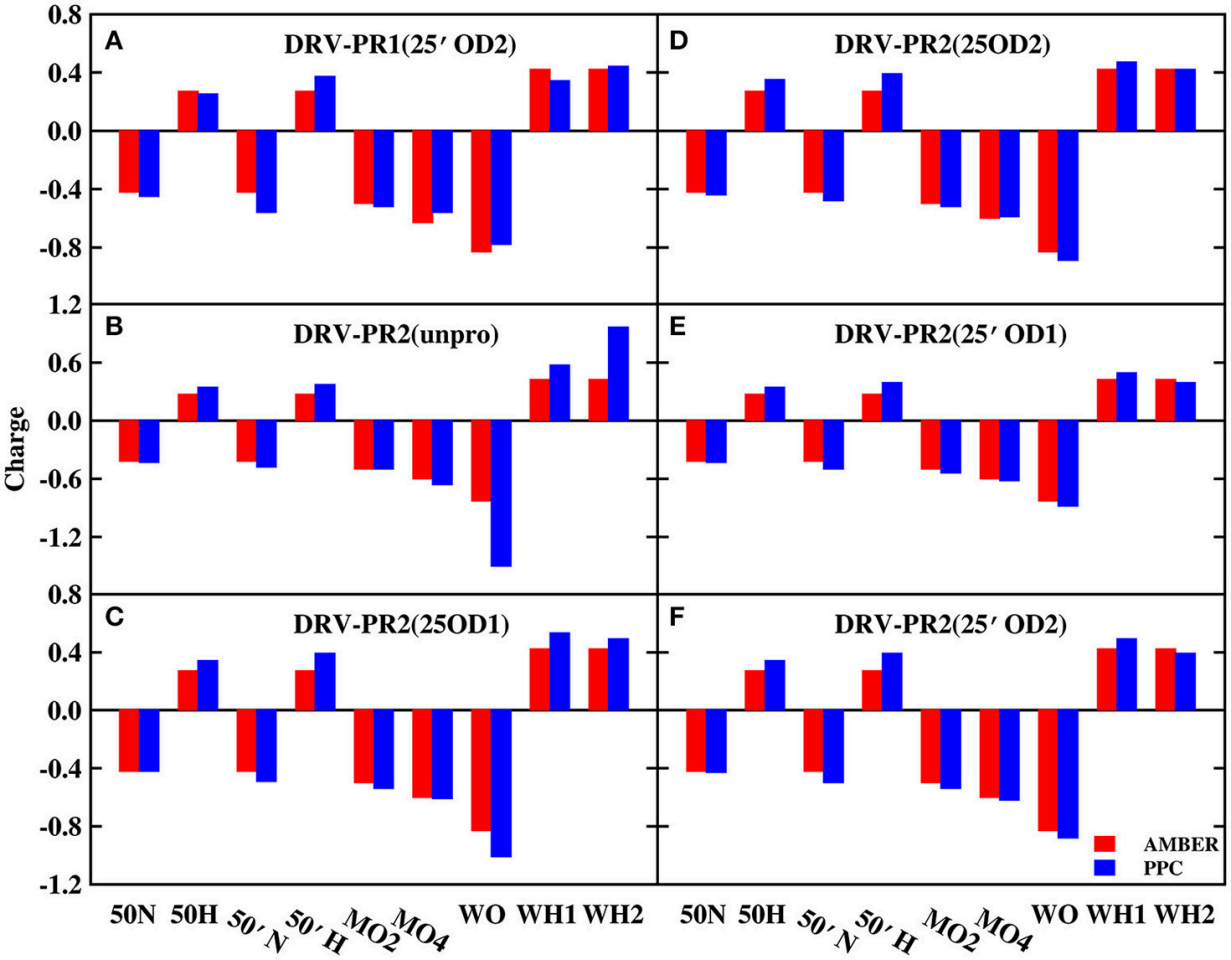
The charge of bridging water molecules and surrounding atoms that form hydrogen bonds in DRV complex. MO2, MO4, and MO5 represent O2, O4, and O5 atoms of ligand molecule, respectively. WO, WH1, and WH2 represent bridging water molecule. **(A)** DRV-PR1(25′OD2) system. **(B)** DRV-PR2(unpro) system. **(C)** DRV-PR2(25OD1) system. **(D)** DRV-PR2(25OD2) system. **(E)** DRV-PR2(25′OD1) system. **(F)** DRV-PR2(25′OD2) system.

**Figure 3:**
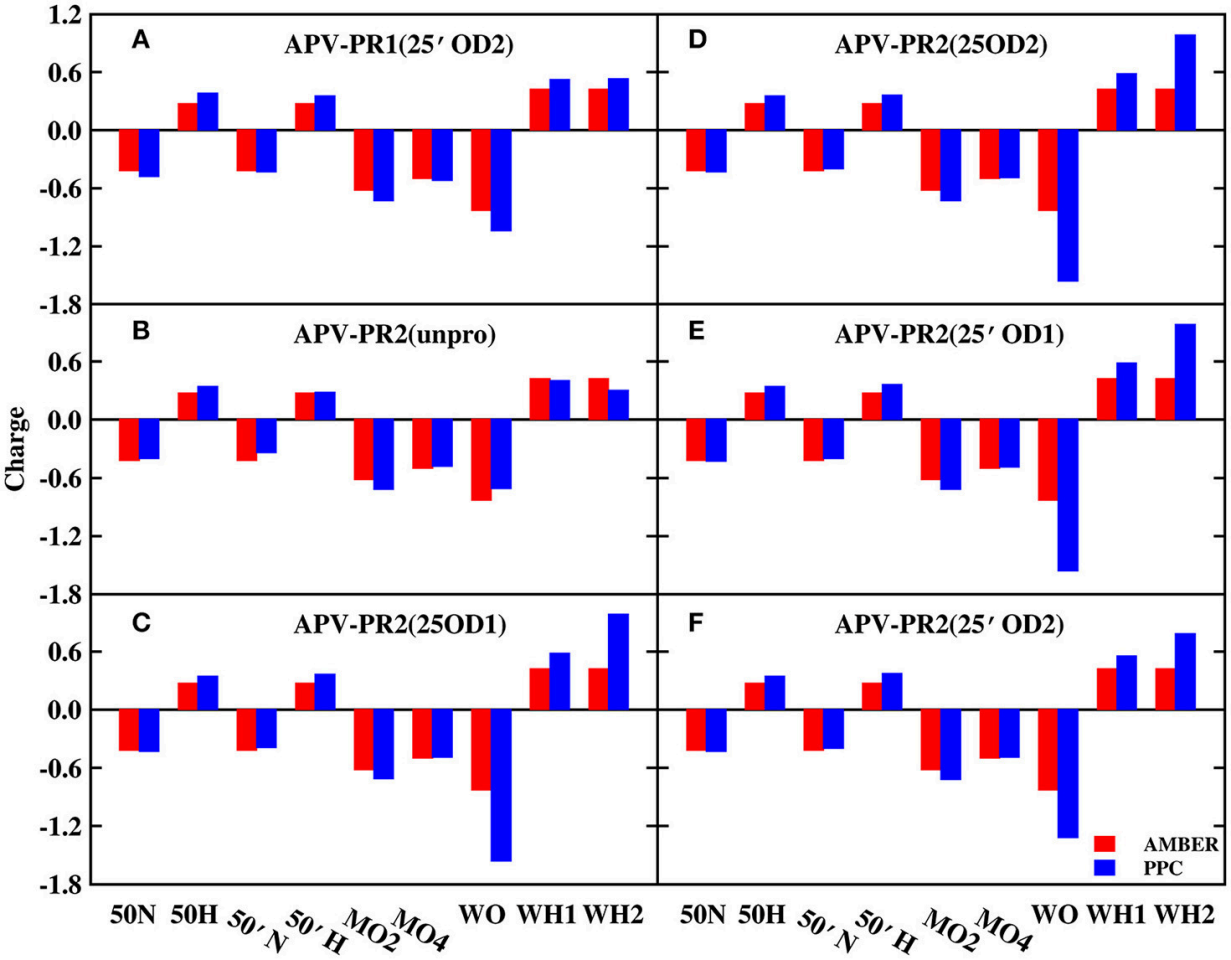
The charge of bridging water molecules and surrounding atoms that form hydrogen bonds in APV complex. **(A)** APV-PR1(25′OD2) system. **(B)** APV-PR2(unpro) system. **(C)** APV-PR2(25OD1) system. **(D)** APV-PR2(25OD2) system. **(E)** APV-PR2(25′OD1) system. **(F)** APV-PR2(25′OD2) system.

In order to explore the reason, those charges of bridging water molecules and the surrounding atoms that form hydrogen bonds with W301 are plotted on Figures 2, 3 under AMBER and PPC force field respectively. For most atoms, the absolute values of charges from PPC are larger than corresponding AMBER charges. The larger polarized charges can provide more attractive electrostatic interaction under PPC than AMBER force field, which results into the strong hydrogen bonds for the binding of protease-W301-inhibitor. Thus, the bridging water is very stable under PPC simulation. The phenomenon may be an excellent illustration that correctly including the polarization effect in MD simulation can give stable and reliable electrostatic environment and therefore it is of critical importance in PR-W301-inbihitor interaction.

### Protonation states of PR2

Considering that the two residues (Asp25 and Asp25′) containing charged groups are very close to each other, protonation prevents the two residues from repulsing each other because of strong Coulomb interaction. Different protonation states are validated according to the local environment. In our works, five possible protonated states of PR2 complex are performed to explore the effect of different types of protonation on structure and binding free energy.

Some average geometric parameters of the protonated region during MD simulation are shown in Table 1. Detailed results are presented at Tables S1, S2 in the Supporting Information. The selection of the six parameters (D_1_~D_6_) is consistent with Hou et al. (Hou and Yu, 2007). The unsigned mean error (UME) of the distance and angle is used to measure deviations between simulated structures and the native structures. The average (AVE) of six parameters (D_1_~D_6_) is used to measure average distance among the three (Asp25, Asp25′ and inhibitor). For DRV-PR2 complex, the average distances in the unprotonated state under AMBER and PPC are 4.18 and 3.40 Å, respectively, larger than the average distances (3.27 Å) of crystal structure and most other protonation states. For APV-PR2 complex, the average distances in the unprotonated state under AMBER and PPC are 4.69 and 4.47 Å, respectively, much larger than the average distances (3.00 Å) of crystal structure and other protonation states. The large conformation change comes from the consequence of the strong Asp-Asp repulsion. Therefore, the unprotonated state is not reasonable in our simulation.

**Table 1:** Average values and unsigned mean errors of geometric parameters of the protonated region during MD simulation.

			**Crystal**	**unpro**		**25OD1**		**25OD2**		**25**^**′**^**OD1**		**25**^**′**^**OD2**	

				**AM**	**PPC**	**AM**	**PPC**	**AM**	**PPC**	**AM**	**PPC**	**AM**	**PPC**
AVE	DRV-PR2 (Å)		3.27	4.18	3.40	3.32	3.63	3.55	3.24	3.36	3.32	3.40	3.45
	APV-PR2 (Å)		3.00	4.69	4.47	3.32	3.52	3.37	3.42	3.40	3.22	3.43	3.33
UME	DRV-PR2	D (Å)	0.00	1.15	0.52	0.50	1.10	0.88	0.44	0.81	0.26	0.76	0.86
		A (°)	0.00	25.12	17.79	9.36	32.20	18.54	5.27	29.36	12.82	18.75	21.20
	APV-PR2	D (Å)	0.00	1.69	1.65	0.37	0.87	0.65	0.62	0.91	0.26	1.09	0.69
		A (°)	0.00	24.44	34.24	13.32	30.20	19.69	20.54	27.84	9.34	35.99	22.10

*AM represents AMBER*.

In contrast, these parameters of distance and angle in the 25′OD1 protonated state under PPC force field are the closest crystal structure among these protonated states. For DRV-PR2 complex, the UME of distance parameters in the 25′OD1 protonated state under PPC force field is 0.26, 0.18 Å less than the next closest state. The UME of angle parameters is 12.82° , which is also small enough. Related to APV-PR2 complex, the UMEs of distance and angle parameters in the 25′OD1 protonated state under PPC force field are 0.26 and 9.34°, respectively, 0.11 and 3.98° less than the next closest state. This indicates that the 25′OD1 protonated state under PPC force field provides better geometry structure in the protonated region than other protonated states.

### The difference in binding free energies

In our works, four methods (AMBER-Nmode, AMBER-IE, PPC-Nmode, and PPC-IE) are used to calculate the binding free energy between PR and inhibitor. Under the condition of ensuring the stability of bridging water and the convergence of IE method, the binding free energy and detailed items are calculated and shown in Tables S3, S4 in the Supporting Information. It can be seen that there is considerable difference between binding free energies under different protonated state. This indicates that type of protonation does play an indispensable role in calculation of binding free energy.

**Figure 4:**
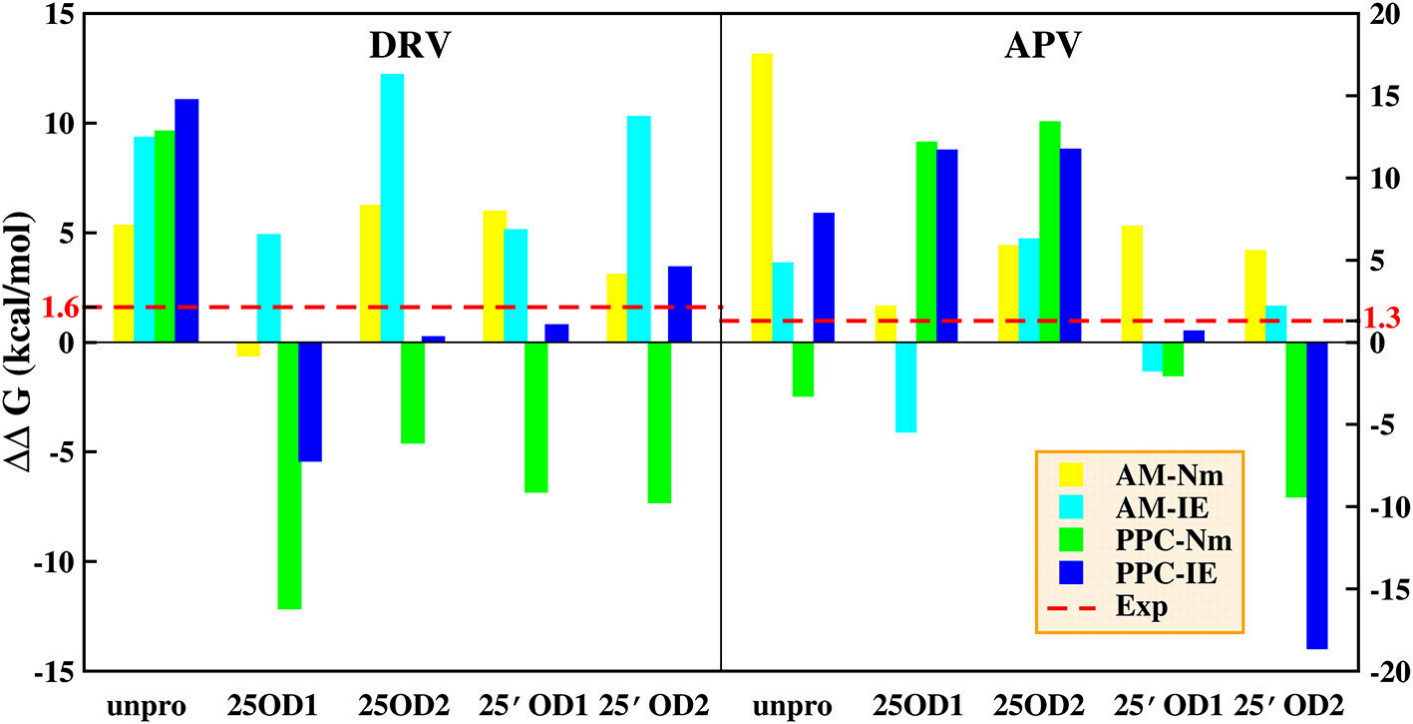
Difference between binding free energy of PR1 complex and binding free energy of PR2 complex. AM represents AMBER; Nm represents Nmode. Cyan lines represent experimental values. *The experimental value is calculated by Weber (Tie et al., 2012).

In order to explore the influences of types of protonation in deep and the calculation method on binding free energy, we calculate the difference of binding free energy between PR1 and PR2 complex. Those results are shown on Table 2 and Figure 4. In Figure 4, red lines represent the difference of binding free energy of between the experimental values of PR1-inhibitor and PR2-inhibitor, which is 1.6 and 1.3 kcal/mol in DRV complexes and APV complexes, respectively. In DRV complexes, the difference of binding free energy between PR1-DRV and PR2-DRV calculated by PPC-IE method in the 25′OD1 protonated state in PR2 system is 0.78 kcal/mol. In APV complexes, it is 0.66 kcal/mol. Both of these two values are the closest to the experimental value among all combinations. And at the above condition, the rank of the predicted binding free energies is significantly consistent with the experimental rank. This indicates that binding free energy calculated by PPC-IE method in the 25′OD1 protonated state in PR2 system is the optimal choice in our study.

**Table 2:** Binding free energy and delta of PR1 and PR2 complexes.

	**AM-Nmode**		**AM-IE**		**PPC-Nmode**		**PPC-IE**		***ΔG***_exp_*	

	**Mean**	**Delta**	**Mean**	**Delta**	**Mean**	**Delta**	**Mean**	**Delta**	**Mean**	**Delta**
DRV-PR1 (25′OD2)	−27.17		−31.27		−24.52		−29.32		−16.0	
DRV-PR2 (unpro)	−21.86	5.31	−21.95	9.32	−14.91	9.61	−18.27	11.05		
DRV-PR2 (25OD1)	−27.78	−0.61	−26.38	4.89	−36.78	−12.26	−34.74	−5.42		
DRV-PR2 (25OD2)	−20.94	6.23	−19.07	12.20	−29.11	−4.59	−29.09	0.23		
DRV-PR2 (25′OD1)	−21.21	5.96	−26.15	5.12	−31.35	−6.83	−28.54	0.78	−14.4	1.6
DRV-PR2 (25′OD2)	−24.10	3.07	−20.99	10.28	−31.84	−7.32	−25.91	3.41		
APV-PR1 (25′OD2)	−26.65		−22.58		−15.95		−15.19		−13.0	
APV-PR2 (unpro)	−9.15	17.50	−17.77	4.81	−19.23	−3.28	−7.37	7.82		
APV-PR2 (25OD1)	−24.48	2.17	−28.02	−5.44	−3.82	12.13	−3.53	11.66		
APV-PR2 (25OD2)	−20.81	5.84	−16.34	6.24	−2.57	13.38	−3.47	11.72		
APV-PR2 (25′OD1)	−19.61	7.04	−24.31	−1.73	−17.99	−2.04	−14.53	0.66	−11.7	1.3
APV-PR2 (25′OD2)	−21.09	5.56	−20.41	2.17	−25.35	−9.40	−33.80	−18.61		

*AM represents AMBER. All values are in kcal/mol*.

* *The experimental value is calculated by Weber (Tie et al., 2012)*.

In view of the consistency of conformation and binding free energy between experimental measure and theoretical calculation, the 25′OD1 protonation model and PPC force field are used to further study.

According to Table 2, it can be observed that the computed binding free energies of DRV complexes are stronger than APV complexes in two PRs consistently. The binding free energies and detailed items of DRV and APV complexes are compared on Table 3. The *G*_*ele*+*pol*_ item contains the electrostatic interactions and polar solvation free energies. In PR1 complexes, the delta of binding free energies between APV-PR1 and DRV-PR1 is 14.13 kcal/mol. The most contribution of the difference comes from vdW interaction (delta is 6.58 kcal/mol). In PR2 complexes, the delta of binding free energies is 14.01 kcal/mol. Although those deltas of electrostatic interaction and polar solvation free energy is special significant, the two offset each other. The total delta of two is 1.33 kcal/mol, which is small enough. The most contribution still comes from vdW interaction (delta is 12.89 kcal/mol).

**Table 3:** Binding free energy and delta of DRV and APV complexes under PPC force field.

	**PR1**			**PR2**		
	**DRV**	**APV**	**Delta**	**DRV**	**APV**	**Delta**
Δ*E*_*ele*_	−90.12	−88.40	1.72	−93.99	−115.06	−21.07
Δ*E*_*vdw*_	−62.72	−56.14	6.58	−63.59	−50.70	12.89
Δ*G*_*pol*_	105.69	109.51	3.82	107.72	130.12	22.40
Δ*G*_*nopol*_	−6.95	−6.89	0.06	−7.03	−6.86	0.17
Δ*G*_*ele*+*pol*_	15.57	21.11	5.54	13.73	15.06	1.33
−*TΔS*(*IE*)	24.78	26.73	1.95	28.35	27.97	−0.38
Δ*G*_*bind*_	−29.32	−15.19	14.13	−28.54	−14.53	14.01

*PR2 complexes adopt 25′OD1 protonation model. Entropy change is calculated by IE*.

**Figure 5:**
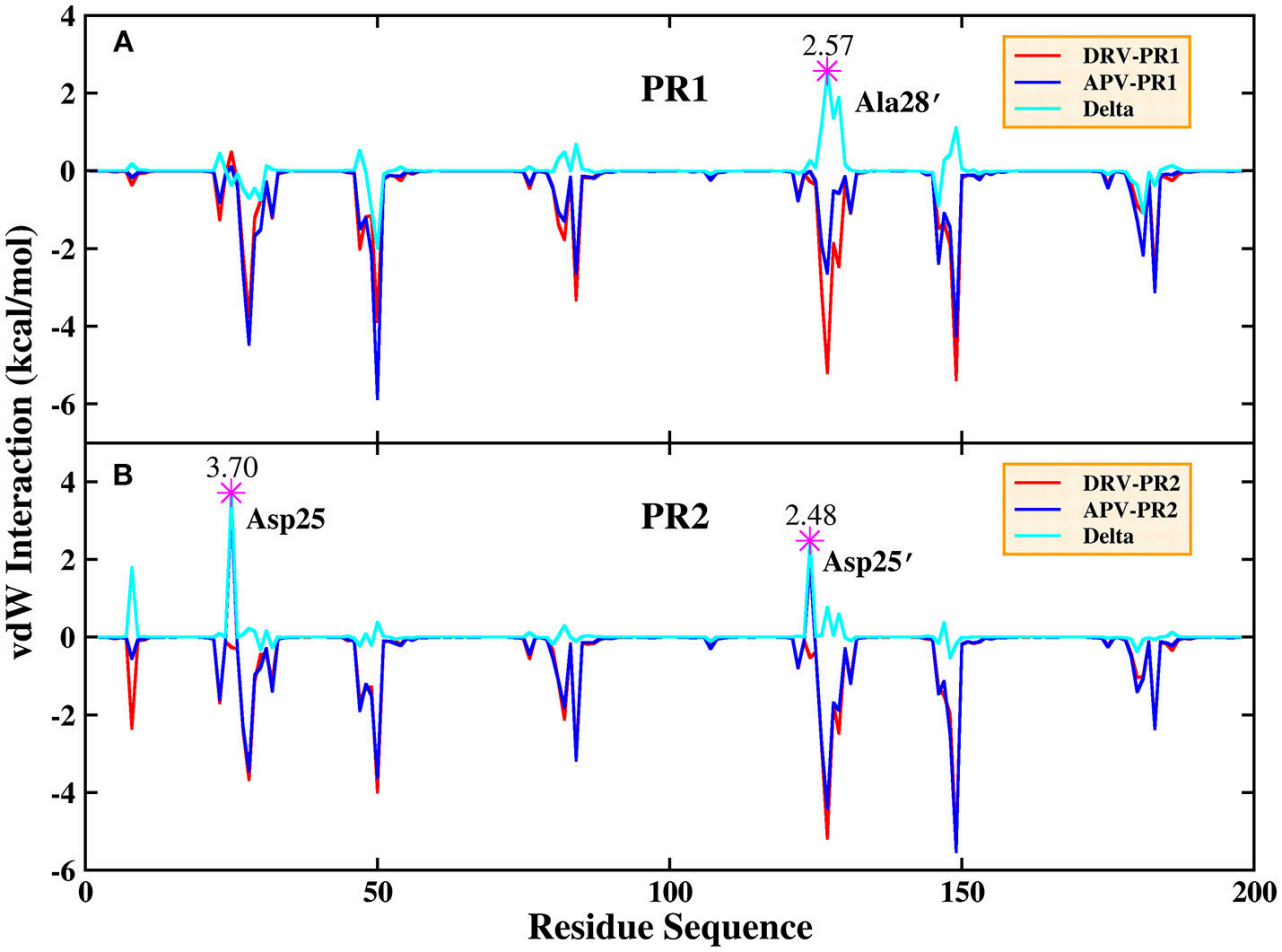
Decomposition of residue based on vdW interaction. **(A)** PR1 complexes. **(B)** PR2 complexes.

**Figure 6:**
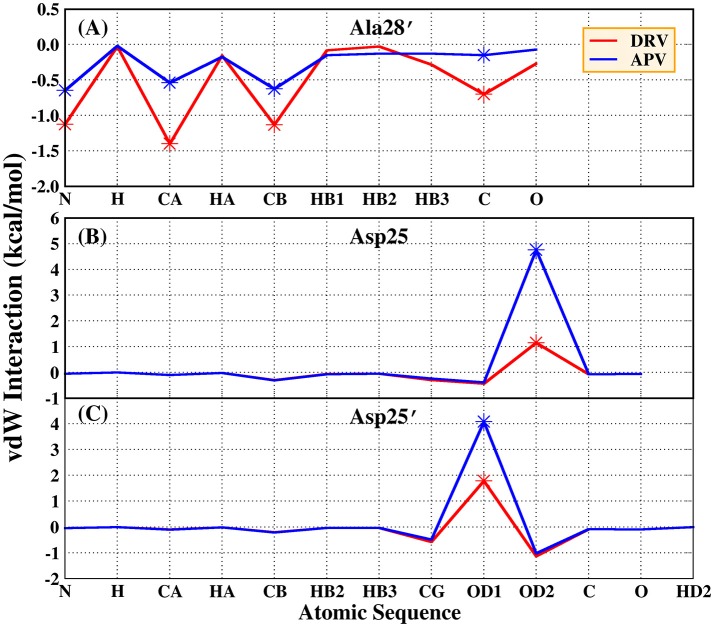
vdW interaction of per atom and inhibitor in Ala28′ of PR1 and Asp25/Asp25′ of PR2. **(A)** Ala28′ residues in PR1. **(B)** Asp25 residue in PR2; **(C)** Asp25′ residues in PR2.

**Figure 7:**
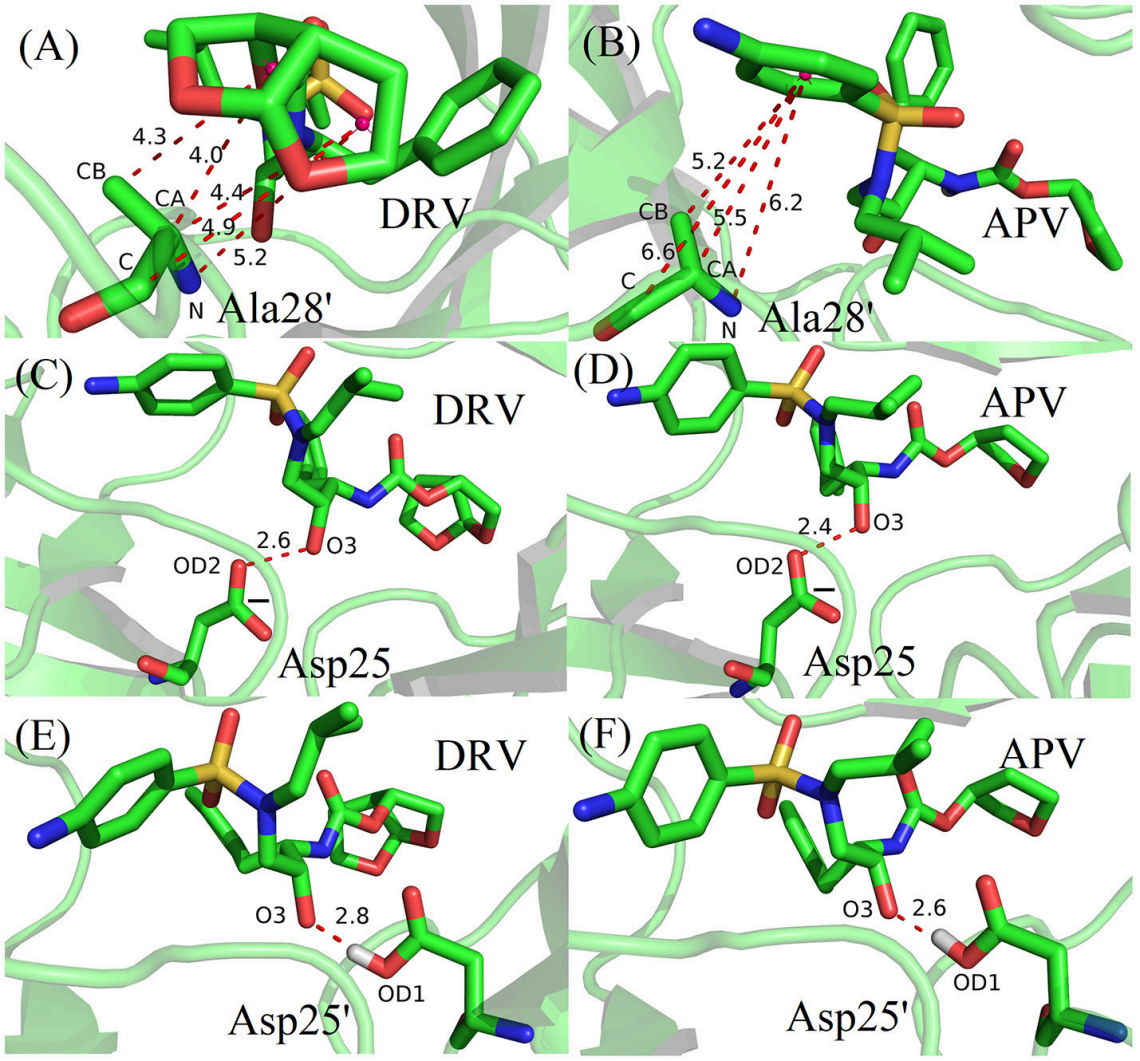
The geometrical positions of Ala28′, Asp25/Asp25′ and two inhibitors based on the lowest potential energy structure from MD trajectory. **(A)** Ala28′ residue in DRV-PR1. **(B)** Ala28′ residue in APV-PR1. **(C)** Asp25residue in DRV-PR2. **(D)** Asp25 residue in APV-PR2. **(E)** Asp25′ residue in DRV-PR2. **(F)** Asp25′ residue in APV-PR2.

Based on vdW interaction, the decomposition of residue is performed which is shown in Figure 5. According to Figure 5A, the vdW interaction of Ala28′ in DRV-PR1 complex is more favorable than in APV-PR1 complex with the delta value of 2.57 kcal/mol. Subsequently, the vdW interaction of per atom of Ala28′ and inhibitor is calculated and shown in Figure 6A. It can be observed that the difference of vdW interaction is mainly reflected on N, CA, CB, and C atoms. Figures 7A,B depict the geometrical positions of Ala28′ and two inhibitors based on the lowest potential energy structure from MD trajectory. These distances between above atoms and hydrophobic groups of DRV are smaller than APV, which result in stronger vdW interaction. The structural descriptions agree well with analysis of vdW interaction.

According to Figure 5B, the vdW interaction of Asp25 and Asp25′ in APV-PR2 complex is more unfavorable than DRV-PR2. Those deltas are 3.70 and 2.48 kcal/mol, respectively. The vdW interaction of per atom of Asp25/Asp25′ and inhibitor is calculated and shown in Figures 6B,C, respectively. The difference of vdW interaction is mainly reflected on OD2/OD1 atom in Asp25/Asp25′, respectively. Figures 7C–F depict the geometrical positions of Asp25/Asp25′ and two inhibitors based on the lowest potential energy structure from MD trajectory. These distances between OD2/OD1 atom and O3 atom of APV are smaller than in DRV complex. Excessively short distances result into stronger repulsion among these atoms. The vdW interaction OD2/OD1 atom and O3 atom in Asp25-DRV, Asp25-APV, Asp25′-DRV, and Asp25′-APV is 1.35, 5.00, 2.43, and 4.72 kcal/mol, respectively. The structural descriptions and analysis of vdW interaction are both consistent.

### Bridging water molecule and hydrogen bond network

Water molecules play a critical role in regulating the structure and function of proteins and maintaining the fundamental biological process of cells (Chaplin, 2006; Vukovic et al., 2016). Philip Ball called water “an active matrix of life for cell and molecular biology” (Ball, 2017). In this paper, the bridging water molecules and their hydrogen bond networks are analyzed to explore its impact on the binding of PR and inhibitors.

The above calculation on the binding free energy has included the effect of the bridging water W301 by considering it as part of PR. Next, in order to investigate the contribution of W301, we again calculate the binding free energy excluding the bridging water. These energies of individual items are compared with the previous energy calculated in the presence of bridging water and are shown in Table 4. Obviously, the electrostatic interaction and polar solvation free energy undergo significant changes before and after considering the bridging water. In particular, the electrostatic interaction makes strong and favorable contribution toward binding free energy at the presence of the W301. The reason is mainly from the four hydrogen bonds formed by bridging water. In PR1 complexes, the contribution of bridging water to total binding energy is −3.43 and −6.62 kcal/mol, respectively. This indicates that bridging water does play a powerful role in promoting the binding of the two PR1 complexes which is significantly consistent with other studies (Lu et al., 2006; Barillari et al., 2007; Duan et al., 2007). However, in PR2 complexes, the contribution of bridging water to total binding energy is 0.12 kcal/mol and 2.06 kcal/mol, respectively. This observation clearly shows that bridging water not only doesn't promote the binding of PR2 complexes, but also plays an inhibitory role.

**Table 4:** Comparison of binding free energy with bridging water (WAT) and no bridging water (NW) when using the PPC-IE method in the 25′OD1 protonated state.

	**DRV-PR1 (25**^**′**^**OD2)**			**DRV-PR2 (25**^**′**^**OD1)**			**APV-PR1 (25**^**′**^**OD2)**			**APV-PR2 (25**^**′**^**OD1)**		
	**NW**	**WAT**	**Delta**	**NW**	**WAT**	**Delta**	**NW**	**WAT**	**Delta**	**NW**	**WAT**	**Delta**
Δ*E*_*ele*_	−81.52	−90.12	−8.60	−79.58	−93.99	−14.41	−70.17	−88.40	−18.23	−85.67	−115.06	−29.39
Δ*E*_*vdw*_	−62.95	−62.72	0.23	−64.79	−63.59	1.20	−59.13	−56.14	2.99	−55.70	−50.70	5.00
Δ*G*_*pol*_	101.28	105.69	4.41	97.39	107.72	10.33	100.21	109.51	9.30	100.51	130.12	29.61
Δ*G*_*nopol*_	−6.94	−6.95	−0.01	−7.02	−7.03	−0.01	−6.88	−6.89	−0.01	−6.85	−6.86	−0.01
Δ*G*_*ele*+*pol*_	19.76	15.57	−4.19	17.81	13.73	−4.08	30.04	21.11	−8.93	14.84	15.06	0.22
−*TΔS*(*IE*)	24.24	24.78	0.54	25.35	28.35	3.00	27.40	26.73	−0.67	31.12	27.97	−3.15
Δ*G*_*bind*_	−25.89	−29.32	−3.43	−28.65	−28.54	0.11	−8.57	−15.19	−6.62	−16.59	−14.53	2.06

*All values are in kcal/mol*.

**Figure 8:**
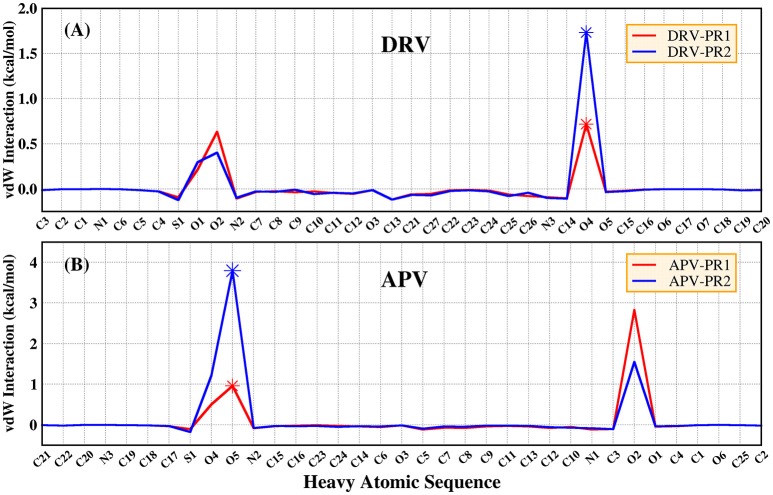
The vdW interaction of bridging water and per heavy atom of inhibitors. **(A)** DRV complexes. **(B)** APV complexes.

**Figure 9:**
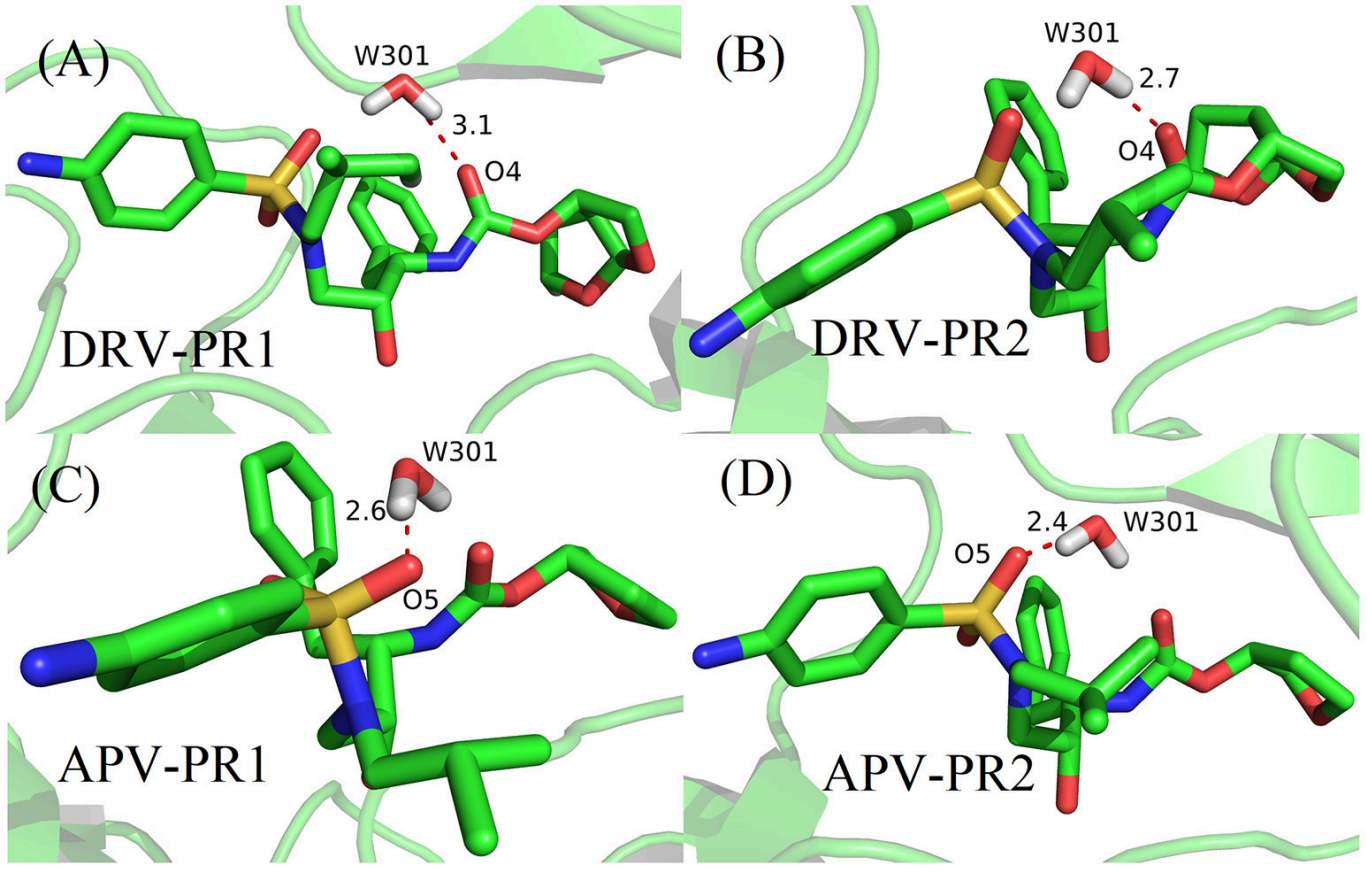
The geometrical positions of bridging water and inhibitors based on the lowest potential energy structure from MD trajectory. **(A)** DRV-PR1 complexes. **(B)** DRV-PR2 complexes. **(C)** APV-PR1 complexes. **(D)** APV-PR2 complexes.

According to Table 4, considering that the contribution of polar solvation free energy to delta is cancelled by electrostatic interactions, vdW interaction plays a major contribution to delta of binding free energy. In our study, bridging water is treated as part of protease. Therefore, the vdW interaction of bridging water and per heavy atom of inhibitors is further calculated and they are shown in Figure 8. It can be clearly observed that vdW interaction between O4 atom of DRV and bridging water showed a stronger unfavorable contribution in PR2 complex than PR1 complex. Similarly, O5 atom of APV obtains the same conclusion as above, as well. Subsequently, the geometrical positions of bridging water and inhibitors in four complexes based on the lowest potential energy structure are depicted in Figure 9. Obviously, these distances between O4/O5 atom of inhibitors and bridging water are smaller in PR2 complexes than PR1 complexes. Excessively short distances lead to stronger repulsion between bridging water and inhibitors. The different geometrical positions may be from the binding environment where the bridging water molecules are located. It will further be analyzed in the analysis of the binding pocket section. To some extent, the unfavorable contribution of bridging water explains the reason for the decline in the effectiveness of PR2-inbibitor binding.

Based on the above analysis, the electrostatic interaction makes strongly favorable contribution toward binding free energy. The main reason is from the four hydrogen bonds formed by bridging water molecules. In the next step, the analysis of hydrogen bond network of protease-W301-inhibitor is performed. Among the four hydrogen bonds, two are attached to the inhibitor and two are attached to the residues Ile50 and Ile50′. These two residues are located in the flap A and flap B tip, respectively. They control the substrate to enter or leave the substrate-binding cavity by closing or opening pocket of cavity. This means that the hydrogen bond network not only enhances electrostatic interactions, but also affects the binding of proteins and inhibitors by changing the structure and function of the binding pocket.

In our works, the average distance, angle and occupancy of each hydrogen bond during MD simulation are calculated and shown on Table 5. Of the 16 hydrogen bonds, the average distance of 13 hydrogen bonds is less than 3.5 Å, the average angle of all hydrogen bonds is greater than 120°, and the occupancy of 10 hydrogen bonds is greater than 80%. Moreover, each bridging water forms at least two stable hydrogen bonds connected to proteins and inhibitor, respectively. The above data shows the vast majority of hydrogen bonds are very stable during the simulation. This result explains the significant changes in electrostatic interactions before and after considering the bridging water.

**Table 5:** Occupancy of hydrogen bonds of bridging water during MD simulation.

**System**	**Acceptor**	**Donor**	**Distance**	**Angle**	**Occupancy**
			**(Å)**	**(^°^)**	**(%)**
DRV-PR1 (25′OD2)	WATO	Ile50N-H	3.56	134.39	44.41
	WATO	Ile50′N-H	2.91	164.83	99.80
	DRVO4	WATO-H1	2.88	159.27	99.22
	DRVO2	WATO-H2	2.99	131.83	65.35
DRV-PR2 (25′OD1)	WATO	Ile50N-H	3.68	135.10	39.28
	WATO	Ile50′N-H	2.87	164.79	100.00
	DRVO4	WATO-H1	2.75	151.58	86.96
	DRVO2	WATO-H2	3.05	135.66	73.84
APV-PR1 (25′OD2)	WATO	Ile50N-H	2.83	166.81	99.96
	WATO	Ile50′N-H	3.56	136.44	47.20
	DRVO2	WATO-H1	2.64	167.29	100.00
	DRVO5	WATO-H2	2.88	148.46	81.96
APV-PR2 (25′OD1)	WATO	Ile50N-H	3.02	161.37	92.14
	WATO	Ile50′N-H	2.82	168.32	100.00
	DRVO2	WATO-H1	2.76	164.83	99.92
	DRVO5	WATO-H2	2.88	157.6	74.84

### Comparison of binding pockets of PR1 and PR2

**Figure 10:**
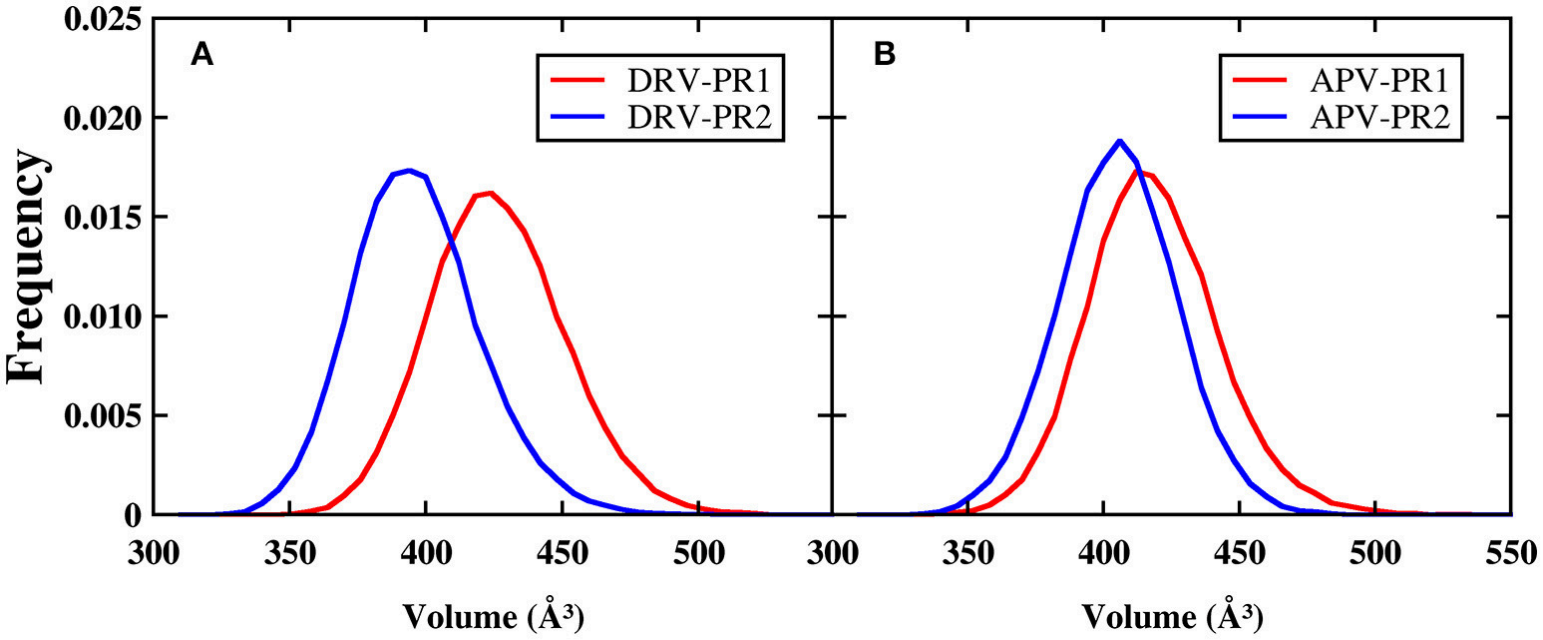
The frequency distributions of volume of the binding pockets during MD simulation. The center of the binding pocket is set as the centroid of the inhibitor. **(A)** DRV complexes. **(B)** APV complexes.

In order to explore the mechanism of binding affinity of inhibitor to PR2, it is essential to research comprehensively operating mode of binding pocket (Duan et al., 2015; Triki et al., 2018). As the active region of binding between protein and inhibitor, differences of binding pockets can explain essentially the reasons for the decline in the binding effectiveness of PR2 complex. The volume of binding pocket is calculated firstly through the POVME (Durrant et al., 2011, 2014) program during MD simulation. The frequency distributions of volume calculated are plotted in Figure 10. It is obviously observed that volumes of binding pocket of PR2 are both smaller compared with PR1. The average volumes of binding pocket calculated during MD simulation are 423.84, 394.00, 415.92, and 402.98 Å^3^ in DRV-PR1, DRV-PR2, APV-PR1, and APV-PR2 complexes, respectively. Our analysis shows that the significant difference of volume is mainly caused by the two possible factors: one is the difference of volume in native structure between PR1 and PR2; the other is the variation in the flexibility of residues during MD simulation. The volumes of native crystal structure are 385, 368, 353, and 360 Å^3^, respectively, and the influence can be eliminated by calculating the delta of native volume and average volumes. Larger delta of volume indicates greater flexibility of residues during MD simulation. The delta calculated are 38.84, 26.00, 62.92, and 42.98 Å^3^ in DRV-PR1, DRV-PR2, APV-PR1, and APV-PR2 complexes, respectively. Obviously, the delta of volume in PR2 complex is still less than PR1 complex. The result indicates flexibility of residues in PR1 complex is greater during MD simulation than in PR2 complex.

**Figure 11:**
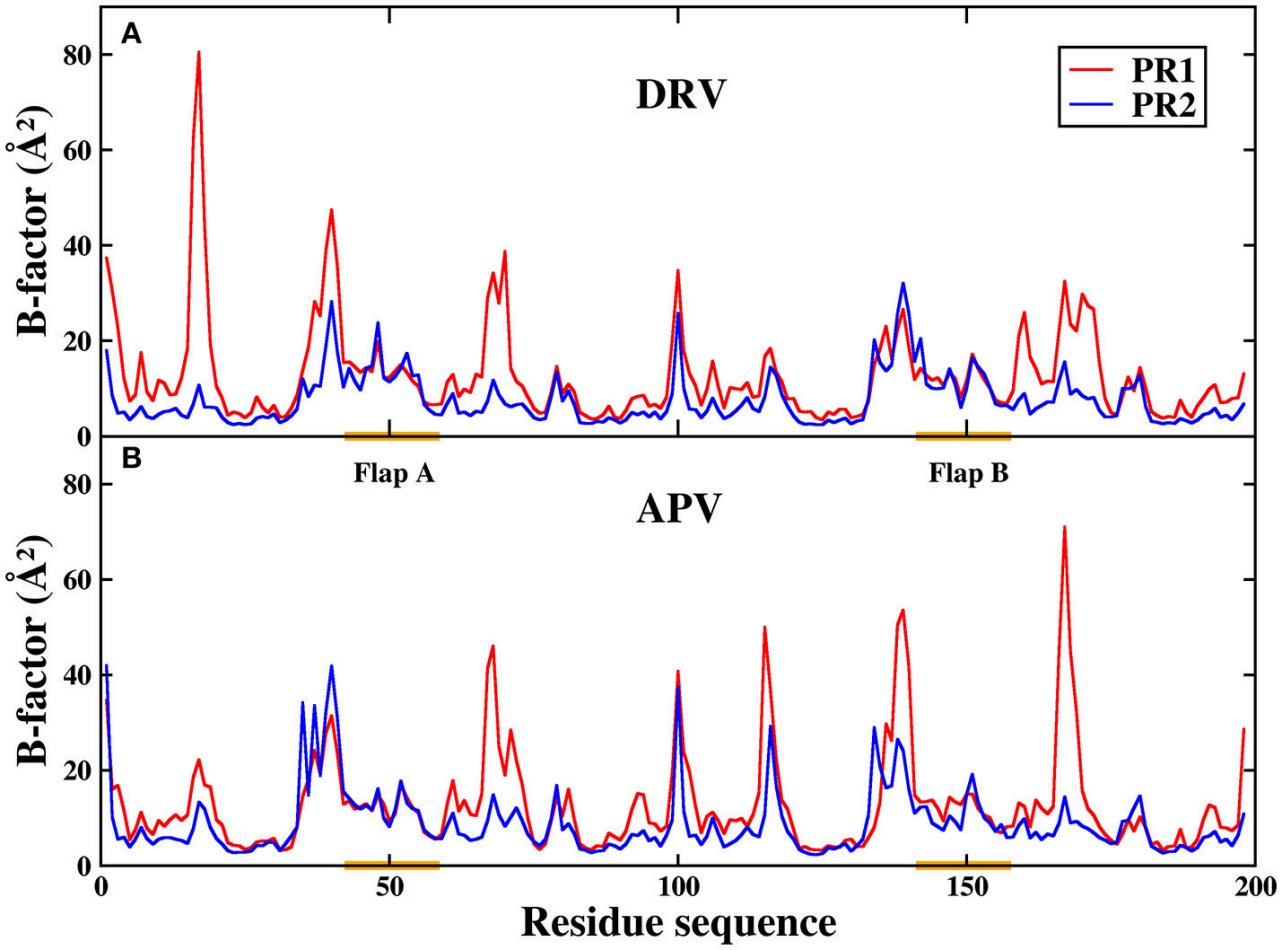
The B-factor of protein Cα atoms in the four complexes during MD simulation. **(A)** DRV complexes. **(B)** APV complexes.

To further analyze residual atomic flexibility, the isotropic temperature factor (B-factor) is utilize to measure atomic fluctuations of individual residues. B-factor reflects the mobility of each residue around its mean position. Figure 11 has plotted the B-factor of protein Cα atoms. The average B-factors of protein Cα in DRV-PR1, DRV-PR2, APV-PR1, and APV-PR2 complexes are 12.87, 7.69, 12.91, and 9.02 Å^2^, respectively. Obviously, according to above result, the B-factors of PR2 complexes are less than that of PR1 complex. Current research illustrates the mobility of PR1 complexes is greater, compared to PR2 complexes. The different behavior patterns of the two proteins explain the difference in the volume of the binding pocket.

**Figure 12:**
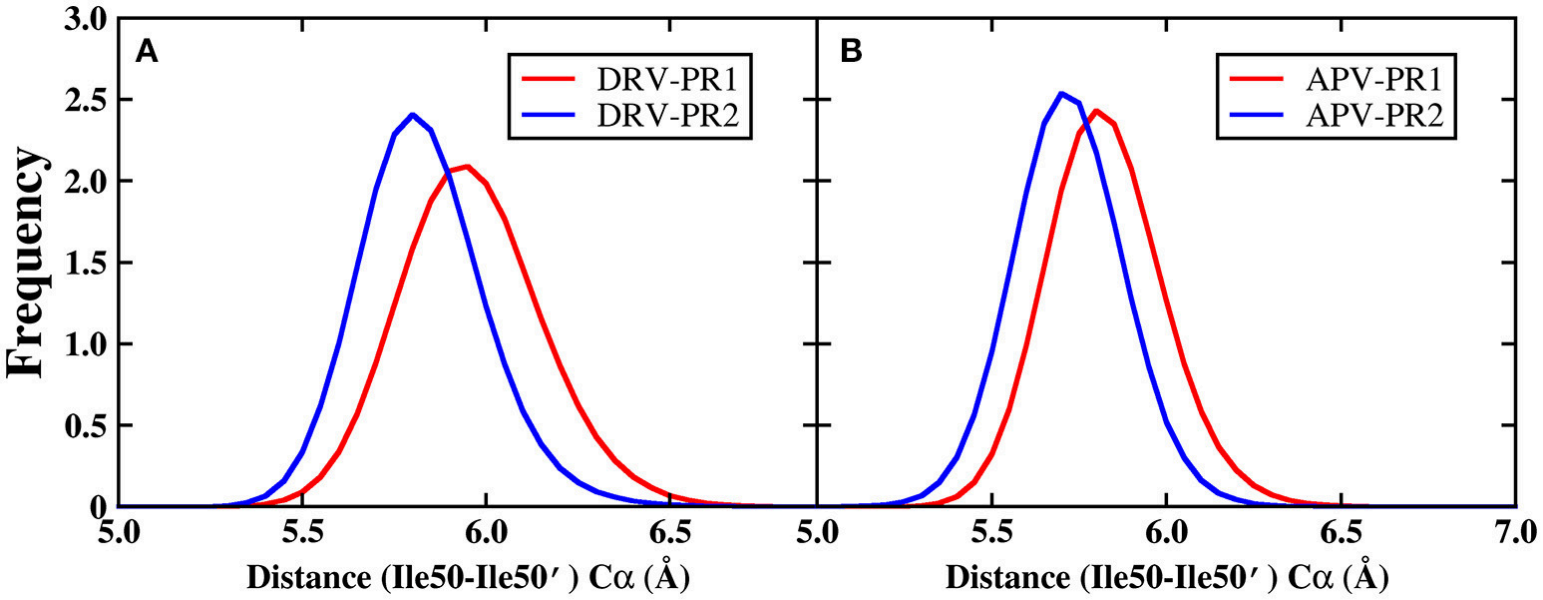
The frequency distribution of distance between Ile50 (Cα) and Ile50′ (Cα) during MD simulation. **(A)** DRV complexes. **(B)** APV complexes.

In addition, Figure 11 shows that the B-factor of flap A and flap B does not differ significantly between PR1 and PR2, relative to other regions. As we all know, flap dynamics not only affects the enzyme catalysis of PR, but also controls the substrate to enter or leave the substrate-binding cavity. Therefore, it is essential to further probe the local structure of this region. In this paper, the distance of the flap tip (Ile50 and Ile50′) is calculated to explore the extent of flap dynamics. The frequency distribution of distance between Ile50 (Cα) and Ile50′ (Cα) is plotted in Figure 12. The average distances are 5.95, 5.80, 5.80, and 5.70 Å in DRV-PR1, DRV-PR2, APV-PR1, and APV-PR2 complexes, respectively. It is clearly seen that the flap area with a tighter structure in PR2 complex than in PR1 complex makes the pocket of cavity tend to close and this results into a smaller volume, which in turn causes local differences of residues lining their surfaces and affects the binding mode of PR2 complex. At the same time, the tighter structure in PR2 complex leads to distances between bridging water and inhibitors shorter and thus produces stronger vdW repulsion than in PR1 complex. This explains further the reason of the unfavorable contribution of bridging water in PR2 complexes.

### The decomposition of residue

Searching for hot-spot residues and exploring its interactions with inhibitors have long been an important step in understanding the binding mechanism of complex. In our work, combining the enthalpy change calculated by MM/GBSA with the entropy change calculated by IE, the binding free energy is decomposed to generate more detailed residue-inhibitor interaction spectrum.

**Figure 13:**
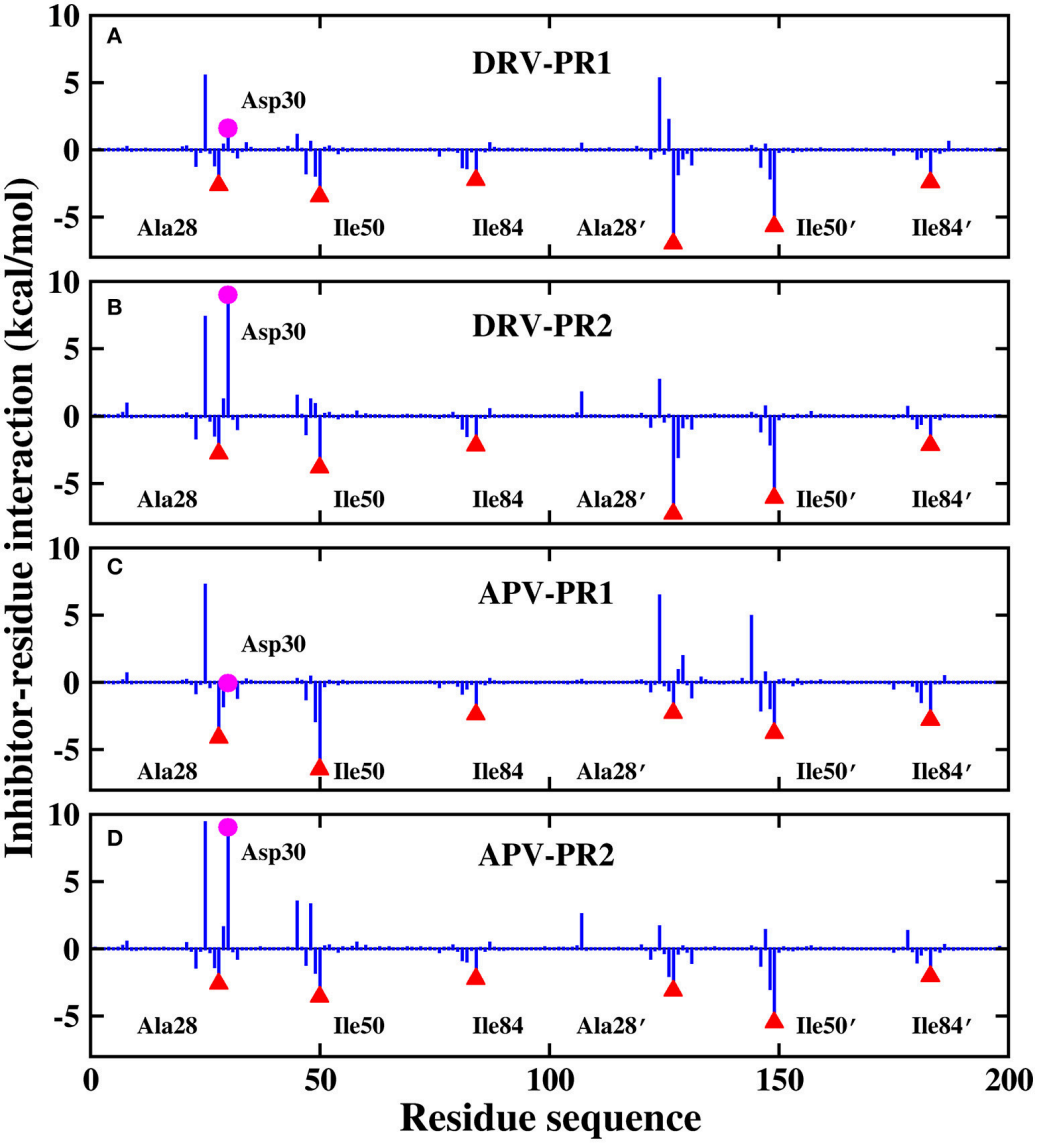
Decomposition of the binding free energy on a per-residue basis. **(A)** DRV-PR1 complexes. **(B)** DRV-PR2 complexes. **(C)** APV-PR1 complexes. **(D)** APV-PR2 complexes.

**Figure 14:**
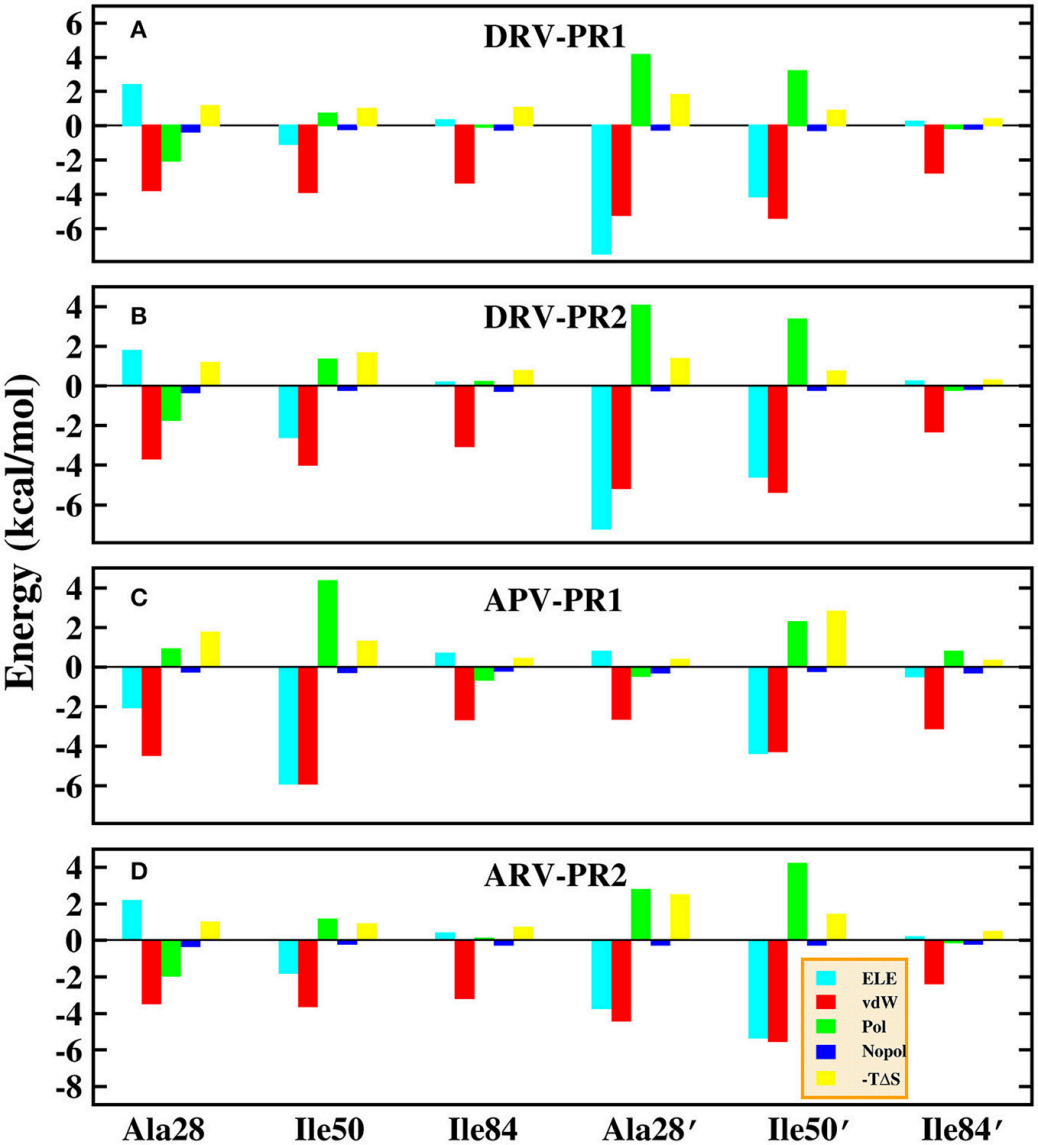
Decomposition of the binding free energy on a per-basis into contributions from electrostatic interaction (ELE), van der Waals interaction (vdW), polar solvation energy (Pol), non-polar solvation energy (Nopol), and entropy change (-TΔS) in the six hot-spot residues. **(A)** DRV-PR1 complexes. **(B)** DRV-PR2 complexes. **(C)** APV-PR1 complexes. **(D)** APV-PR2 complexes.

Those results have been plotted in the Figure 13. In general, the residue-inhibitor interaction spectrums are extremely similar on the four complexes. Among them, regions of binding pocket (Ala28/Ala28′, Ile50/Ile50′, and Ile84/Ile84′ residues) provide major favorable contribution on binding free energy in the four systems. Those energies contributed from those hot-spot residues are greater than −2 kcal/mol. Further, the contribution of these six hot-spot residues is divided into electrostatic interaction, vdW interaction, polar solvation energy, non-polar solvation energy and entropy change. The result of four complexes is plotted in Figure 14. Obviously, the favorable contribution mainly comes from electrostatic interaction and vdW interaction. Among them, vdW interaction provides the most favorable contribution in the range of −2.3 to −5.8 kcal/mol. This result is basically consistent with the previous other research about PR1 (Meher and Wang, 2012).

**Figure 15:**
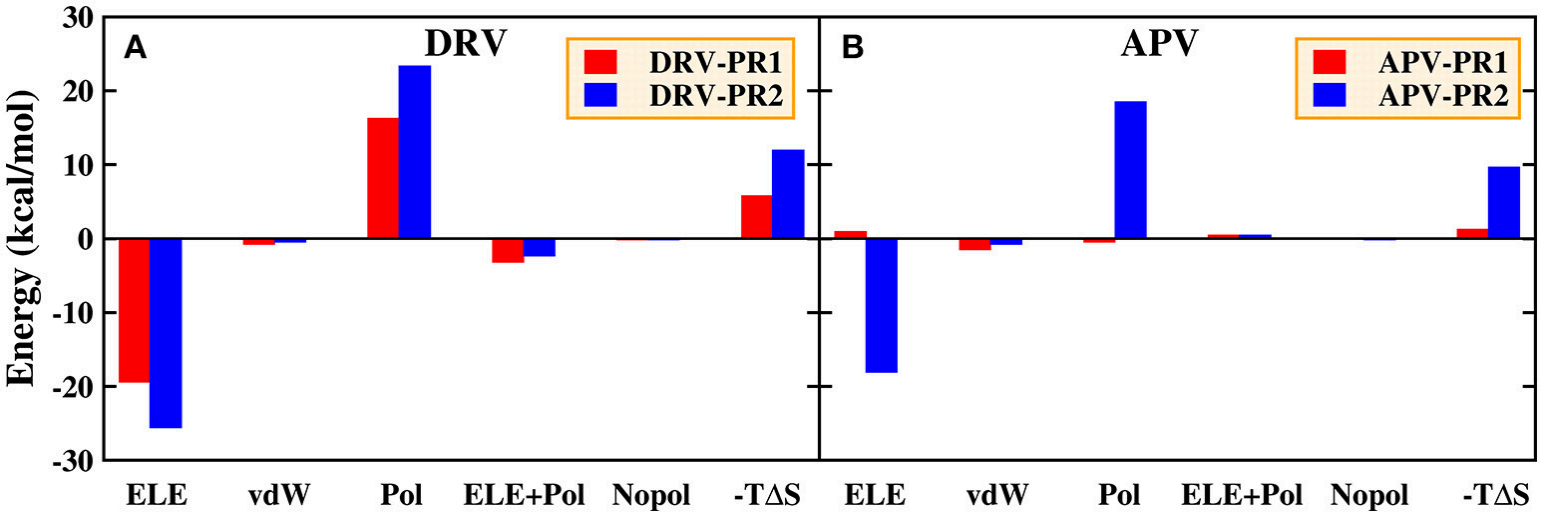
Decomposition of the binding free energy on a per-basis into contributions from electrostatic interaction (ELE), van der Waals interaction (vdW), polar solvation energy (Pol), the sum of electrostatic interaction and polar solvation energy (ELE+Pol), non-polar solvation energy (Nopol), and entropy change (-TΔS) in the Asp30 residues. **(A)** DRV complexes. **(B)** APV complexes.

For the residue-inhibitor interaction spectrum shown in Figure 13, it is worthwhile that Asp30 residue demonstrates great difference on PR1 and PR2 complexes. The energies of inhibitor-residue interaction are 1.60, 8.98, −0.06, and 9.01 kcal/mol in DRV-PR1, DRV-PR2, APV-PR1, and APV-PR2 complexes, respectively. The Asp30 residue contributes unfavorably to the binding of inhibitor against PR2. We further decomposed the binding energy of inhibitor and Asp30 residue for the four complexes shown in Figure 15. Although electrostatic interaction and polar solvation free energy show great differences between PR1 complexes and PR2 complexes. The two offset each other, and the sum of electrostatic interaction and polar solvation free energy is basically same in the two PR systems. The differences are mainly from the entropy change. When DRV is combined with PR1 and PR2, the difference of entropy change is 6.19 kcal/mol. For APV inhibitor, the differences are 8.41 kcal/mol. It may be another reason that causes the decline of the affinity in PR2 complex. The above information is mainly provided by the IE method, which emphasizes the importance of entropy change in the decomposition of residues.

## Conclusions

The current study probes the differences in binding patterns between PR1 and PR2 with inhibitors (DRV and APV) by molecular dynamics simulation. Two force fields, non-polarized traditional AMBER force field and polarized PPC force field, combined two methods for computing entropy change (traditional Nmode and newly developed IE method) are employed to explore the effect of electrostatic polarization on the simulation process and the effectiveness of binding. Our studies find that polarized force field is able to provide a relatively stable binding environment during MD simulation by analysis of stability and protonation states. In addition, polarized force field coupled with the highly efficient IE method shows more significantly consistent with the experimental result in the computation of binding free energy, compared to AMBER force field and Nmode method. Our result shows the following features:

Firstly, five different protonation states of PR2 have been studied in detail through some relevant geometric parameters and the binding free energy calculation. The 25′OD1 protonated state in B chain is found to have the more similar geometry with the crystal structure than other protonated states in two PR2 complexes consistently.
Secondly, the calculated binding free energy is significantly consistent with the experimental observation under the 25′OD1 protonated state in PR2 complexes. Further, the binding free energies of DRV complexes are stronger than APV complexes. The reason is mainly caused by vdW interaction of Ala28′ and Asp25/Asp25′. On the one hand, vdW interaction of Ala28′ in DRV-PR1 complex is more favorable than in APV-PR1 complex; on the other head, the vdW interaction of Asp25 and Asp25′ in APV-PR2 complex is more unfavorable than in DRV-PR2.
Thirdly, we investigate the important impact of bridging water molecule W301 on the effectiveness of binding and our study finds that the bridging water is capable of stably linking proteins and inhibitors through stable hydrogen bonding under PPC force field whether PR1 system or PR2 system. Besides, the bridging water contributes favorably to the binding of two PR1 complexes. However, it is unfavorable to two PR2 complexes. The reason is mainly from that tighter structure of PR2 complex, which results into stronger vdW repulsion between bridging water and inhibiter than that in the PR1 complex. To some extent, it explains the reason for the decline in the effectiveness of inhibitor against PR2.
Fourthly, analysis of the binding pocket finds the B-factor of Cα atoms and distance of flap tip in PR2 complexes all are less than that in PR1 complexes. The poorer flexibility and tighter structure of flap region in PR2 complex make the pocket of cavity closer and the volume smaller than in PR1 complex, which in turn causes local differences of residues lining their surfaces and thus affects the bonding mode of PR2 complex.
Fifthly, the method for the decomposition of residue in traditional MM/GBSA calculation usually neglects entropic contribution due to the difficulty using Nmode method. The IE method is proposed to efficiently compute the residue-specific entropic contribution to PR-inhibitor binding free energy. Using this method, the predicted hot-spot resides (Ala28/Ala28′, Ile50/Ile50′ and Ile84/Ile84′) are found to be nearly similar in the two types PR systems. However, Asp30 residue shows more unfavorable entropic contributions on PR2 complexes, compared with PR1 complexes. The phenomena is hard to obtain using traditional Nmode method. It may be another reason that results in the decline in potency of DRV and APV against PR2 relative to PR1.

We hope that current study will provide essential value and theoretical guidance for the future design of new dual inhibitors targeting HIV proteases.

## Author contributions

YC and YL outperformed the MD simulations, drafted the main text of the manuscript and prepared all the figures. KJ and SZ helped with data analysis. JZ, HL, and LD designed this study and revised the manuscript.

### Conflict of interest statement

The authors declare that the research was conducted in the absence of any commercial or financial relationships that could be construed as a potential conflict of interest.

The reviewer XA and handling Editor declared their shared affiliation.
