# Supplementary material for: Exploring the Reasons for Decrease in Binding Affinity of HIV-2 Against HIV-1 Protease Complex Using Interaction Entropy Under Polarized Force Field
Source: Front Chem. 2018 Aug 24;6:380. doi: 10.3389/fchem.2018.00380 (PMC6117221; doi:10.3389/fchem.2018.00380)
Supplement: Supplementary file 1 [file Table_1.pdf]

Supporting Information

Exploring the Reasons for Decrease in binding affinity of HIV-2 against HIV-1 protease complex using interaction entropy under polarized force field

Yalong Cong [1†], Yuchen Li [1†], Kun Jin [1], Susu Zhong [1], John Z. H. Zhang [2,4,5], Hao Li [1,3*] and Lili Duan [1*]

[1] School of Physics and Electronics, Shandong Normal University, Jinan, China,
[2] Shanghai Engineering Research Center of Molecular Therapeutics and New Drug Development, School of Chemistry and Molecular Engineering, East China Normal University, Shanghai, China,
[3] Department of Science and Technology, Shandong Normal University, Jinan, China,
[4] NYU-ECNU Center for Computational Chemistry at NYU Shanghai, Shanghai, China,
[5] Department of Chemistry, New York University, New York, NY, United States.

Corresponding author (lihao@sdnu.edu.cn; duanll@sdnu.edu.cn)
§ Yalong Cong and Yuchen Li contributed equally to this work

Table S1. Average geometric parameters of the protonated region during MD simulation on the DRV-PR2 complex. D_1 represents distance between inhibitor O3 atom and Asp25OD1 atom; D_2 represents distance between inhibitor O3 atom and Asp25OD2 atom; D_3 represents distance between inhibitor O3 atom and Asp25'OD1 atom; D_4 represents distance between inhibitor O3 atom and Asp25'OD2 atom; D_5 represents distance between Asp25OD1 atom and Asp25'OD1 atom; D_6 represents distance between Asp25OD2 atom and Asp25'OD2 atom; A_1 represents angle formed by Asp25OD1 atom, inhibitor O3 atom and Asp25OD2 atom; A_2 represents angle formed by Asp25OD1 atom, inhibitor O3 atom and Asp25'OD1 atom; A_3 represents angle formed by Asp25'OD2 atom, inhibitor O3 atom and Asp25'OD1 atom; A_4 represents angle formed by Asp25'OD2 atom, inhibitor O3 atom and Asp25OD2 atom; UME represents unsigned mean error.

Table S2. Average geometric parameters of the protonated region during MD simulation on the APV-PR2 complex. The same parameters as in Table S1.

Table S3. Binding free energy and detailed items of DRV-PR complexes. All values are in kcal/mol.

Table S4. Binding free energy and detailed items of APV-PR complexes. All values are in kcal/mol.

Table S1

	Crystal	unpro		25OD1		25OD2		25'OD1		25'OD2	
		AM	PPC	AM	PPC	AM	PPC	AM	PPC	AM	PPC
**D_1 (Å)**	2.97	4.20	3.62	2.79	4.71	3.67	3.21	4.11	3.26	3.18	3.36
**D_2 (Å)**	2.52	3.37	3.46	3.30	3.76	3.60	2.62	2.65	2.75	2.61	2.77
**D_3 (Å)**	2.60	4.87	2.73	3.11	4.00	3.60	3.16	3.68	2.71	3.31	3.42
**D_4 (Å)**	3.20	3.39	2.91	2.58	2.66	2.60	2.57	3.30	3.39	2.68	2.73
**D_5 (Å)**	2.85	4.52	3.09	3.23	2.54	3.57	3.17	3.11	2.96	4.53	4.51
**D_6 (Å)**	5.45	4.75	4.58	4.90	4.09	4.25	4.68	3.33	4.84	4.09	3.89
**AVE (Å)**	3.27	4.18	3.40	3.32	3.63	3.55	3.24	3.36	3.32	3.40	3.45
**UME (Å)**	0.00	1.15	0.52	0.50	1.10	0.88	0.44	0.81	0.26	0.76	0.86
**A_1 (°)**	47.12	31.76	35.98	41.91	27.87	35.70	43.58	27.89	41.51	42.60	40.23
**A_2 (°)**	62.27	59.59	56.08	67.21	32.52	60.86	59.67	47.39	58.60	89.01	83.88
**A_3 (°)**	43.25	17.35	44.79	43.55	30.13	35.84	43.10	36.61	40.80	41.93	40.44
**A_4 (°)**	143.99	87.44	91.71	117.02	77.33	90.06	129.19	67.31	104.45	101.57	90.51
**UME (°)**	0.00	25.12	17.79	9.36	32.20	18.54	5.27	29.36	12.82	18.75	21.20

Table S2

	Crystal	unpro		25OD1		25OD2		25'OD1		25'OD2	
		AM	PPC	AM	PPC	AM	PPC	AM	PPC	AM	PPC
**D_1 (Å)**	2.91	3.62	4.21	2.91	3.98	3.43	3.44	4.17	3.12	2.75	3.14
**D_2 (Å)**	2.34	2.65	2.54	3.36	3.99	2.80	3.10	2.75	2.52	4.25	2.49
**D_3 (Å)**	2.72	5.31	5.84	3.13	4.00	3.18	3.16	3.77	2.58	3.42	3.26
**D_4 (Å)**	2.81	4.62	5.09	2.65	2.69	2.65	2.81	3.34	3.15	3.83	2.63
**D_5 (Å)**	2.37	5.95	4.84	3.01	2.53	3.98	3.75	3.05	2.97	3.30	4.52
**D_6 (Å)**	4.86	5.99	4.32	4.85	3.90	4.17	4.23	3.34	4.95	3.04	3.95
**AVE (Å)**	3.00	4.69	4.47	3.32	3.52	3.37	3.42	3.40	3.22	3.43	3.33
**UME (Å)**	0.00	1.69	1.65	0.37	0.87	0.65	0.62	0.91	0.26	1.09	0.69
**A_1 (°)**	47.89	36.75	24.02	40.72	29.52	40.47	39.05	27.62	43.71	26.10	43.54
**A_2 (°)**	49.72	81.25	54.80	61.04	37.40	77.68	69.93	44.82	61.91	63.41	90.04
**A_3 (°)**	46.61	24.32	21.72	42.65	29.31	41.53	42.18	35.84	44.03	35.14	42.64
**A_4 (°)**	140.98	108.18	57.87	110.15	68.19	102.68	92.31	65.55	122.57	43.96	101.21
**UME (°)**	0.00	24.44	34.24	13.32	30.20	19.69	20.54	27.84	9.34	35.99	22.10

Table S3

System	Method	ΔE_{ele}	ΔE_{vdW}	ΔG_{pol}	ΔG_{nopol}	$-T\Delta S$		ΔG_{bind}		ΔG_{exp} *
						Nmode	IE	Nmode	IE	
**DRV-PR1**	AMBER	-71.56	-65.31	89.10	-7.13	27.73	23.63	-27.17	-31.27	
**(25'OD2)**	PPC	-90.12	-62.72	105.69	-6.95	29.58	24.78	-24.52	-29.32	-16.0
**DRV-PR2**	AMBER	-74.40	-65.43	101.57	-7.28	23.68	23.59	-21.86	-21.95	
**(unpro)**	PPC	-113.10	-62.97	142.81	-7.04	25.39	22.03	-14.91	-18.27	
**DRV-PR2**	AMBER	-68.79	-61.85	90.12	-7.13	19.87	21.27	-27.78	-26.38	
**(25OD1)**	PPC	-99.31	-58.91	105.72	-7.02	22.74	24.78	-36.78	-34.74	
**DRV-PR2**	AMBER	-64.31	-59.96	88.62	-6.99	21.70	23.57	-20.94	-19.07	
**(25OD2)**	PPC	-103.46	-58.78	110.28	-7.00	29.85	29.87	-29.11	-29.09	
**DRV-PR2**	AMBER	-43.88	-61.43	68.66	-6.98	22.42	17.48	-21.21	-26.15	
**(25'OD1)**	PPC	-93.99	-63.59	107.72	-7.03	25.54	28.35	-31.35	-28.54	-14.4
**DRV-PR2**	AMBER	-64.58	-62.31	89.23	-7.07	20.63	23.74	-24.10	-20.99	
**(25'OD2)**	PPC	-85.79	-63.23	101.62	-7.04	22.60	28.53	-31.84	-25.91	

Table S4

System	Method	ΔE_{ele}	ΔE_{vdW}	ΔG_{pol}	ΔG_{nopol}	$-T\Delta S$		ΔG_{bind}		ΔG_{exp} *
						Nmode	IE	Nmode	IE	
**APV-PR1**	AMBER	-64.78	-60.52	81.40	-6.92	24.17	28.24	-26.65	-22.58	
**(25'OD2)**	PPC	-88.40	-56.14	109.51	-6.89	25.97	26.73	-15.95	-15.19	-13.0
**APV-PR2**	AMBER	-45.10	-58.79	75.28	-7.13	26.59	17.97	-9.15	-17.77	
**(unpro)**	PPC	-87.11	-53.26	106.27	-6.82	21.69	33.55	-19.23	-7.37	
**APV-PR2**	AMBER	-58.55	-58.66	76.83	-6.86	22.76	19.22	-24.48	-28.02	
**(25OD1)**	PPC	-64.46	-55.74	97.82	-6.97	25.53	25.82	-3.82	-3.53	
**APV-PR2**	AMBER	-67.16	-56.41	83.78	-6.86	25.84	30.31	-20.81	-16.34	
**(25OD2)**	PPC	-85.11	-49.57	110.76	-6.85	28.20	27.30	-2.57	-3.47	
**APV-PR2**	AMBER	-41.57	-60.29	65.00	-6.84	24.10	19.40	-19.61	-24.31	
**(25'OD1)**	PPC	-115.06	-50.70	130.12	-6.86	24.51	27.97	-17.99	-14.53	-11.7
**APV-PR2**	AMBER	-43.62	-60.12	66.67	-6.89	22.86	23.54	-21.09	-20.41	
**(25'OD2)**	PPC	-107.04	-49.82	111.98	-6.95	26.48	18.03	-25.35	-33.80	

Figure S1. The root-mean-square deviation (RMSD) of the protein backbone atoms relative to the corresponding crystal structure in DRV-PR complexes. (A) the complex of DRV-PR1. (B)~(F) the complexes of DRV-PR2 with five different protonated states.

Figure S2. The root-mean-square deviation (RMSD) of the protein backbone atoms relative to the corresponding crystal structure in APV-PR complexes. (A) the complex of APV-PR1. (B)~(F) the complexes of APVV-PR2 with five different protonated states.

Figure S3. The distances of W301 between inhibitor in DRV-PR complexes during the entire MD simulation.

Figure S4. The distances of W301 between inhibitor in APV-PR complexes during the entire MD simulation.

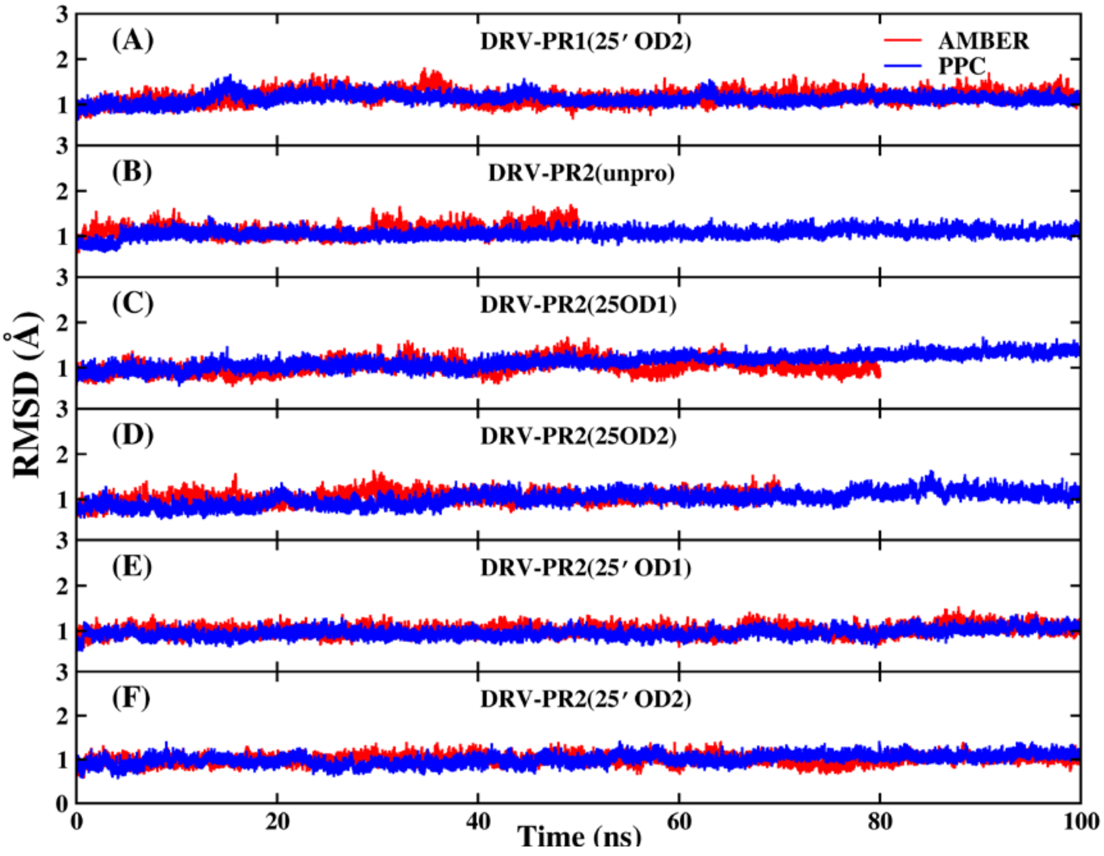

Figure S1

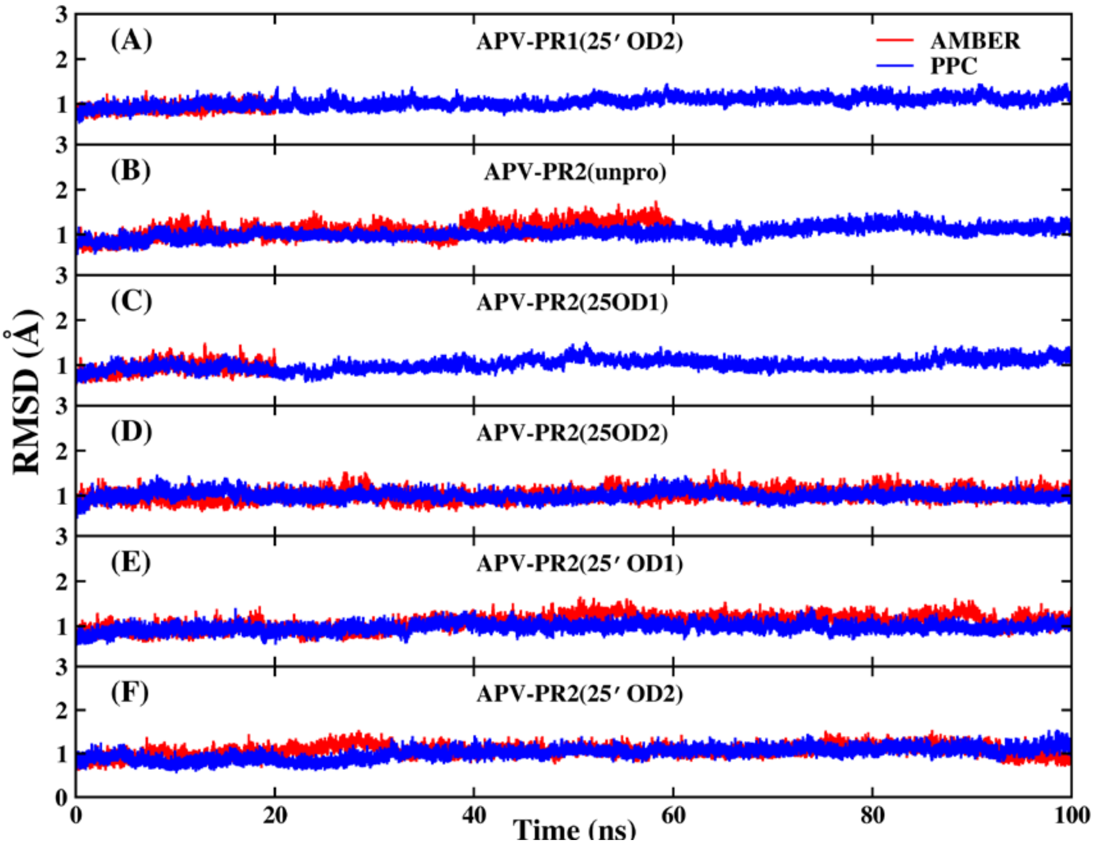

Figure S2

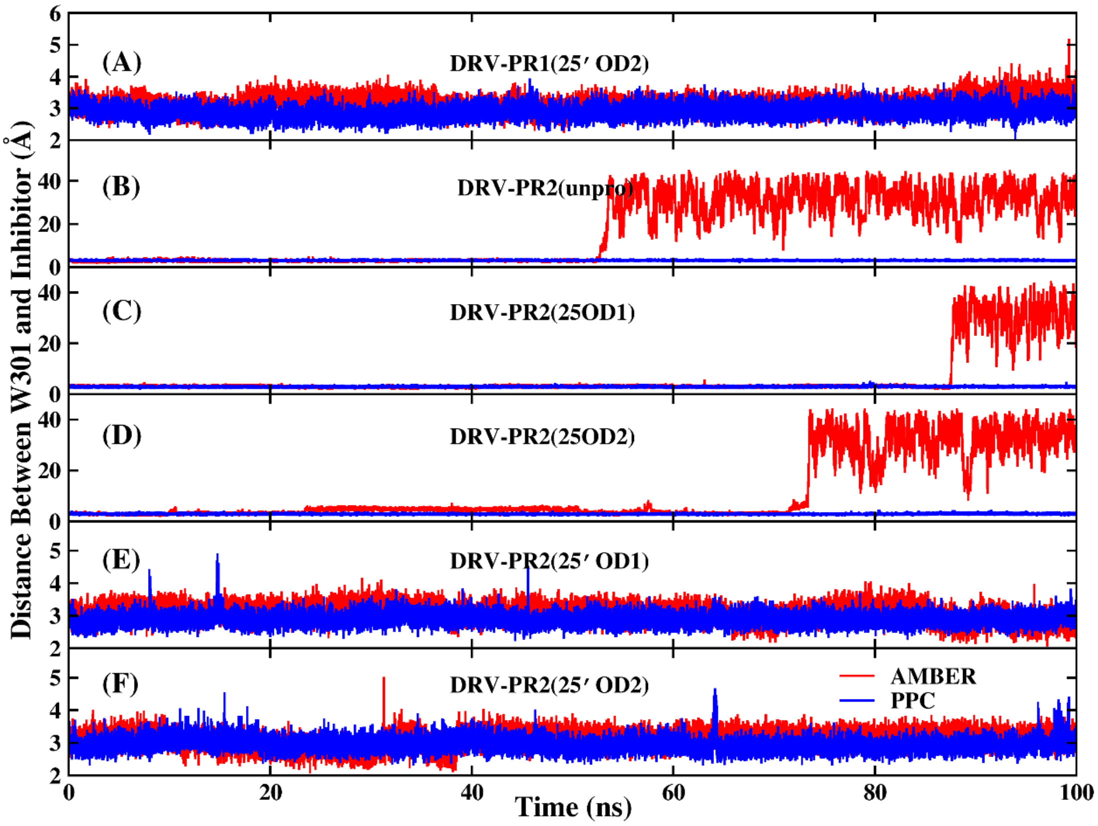

Figure S3

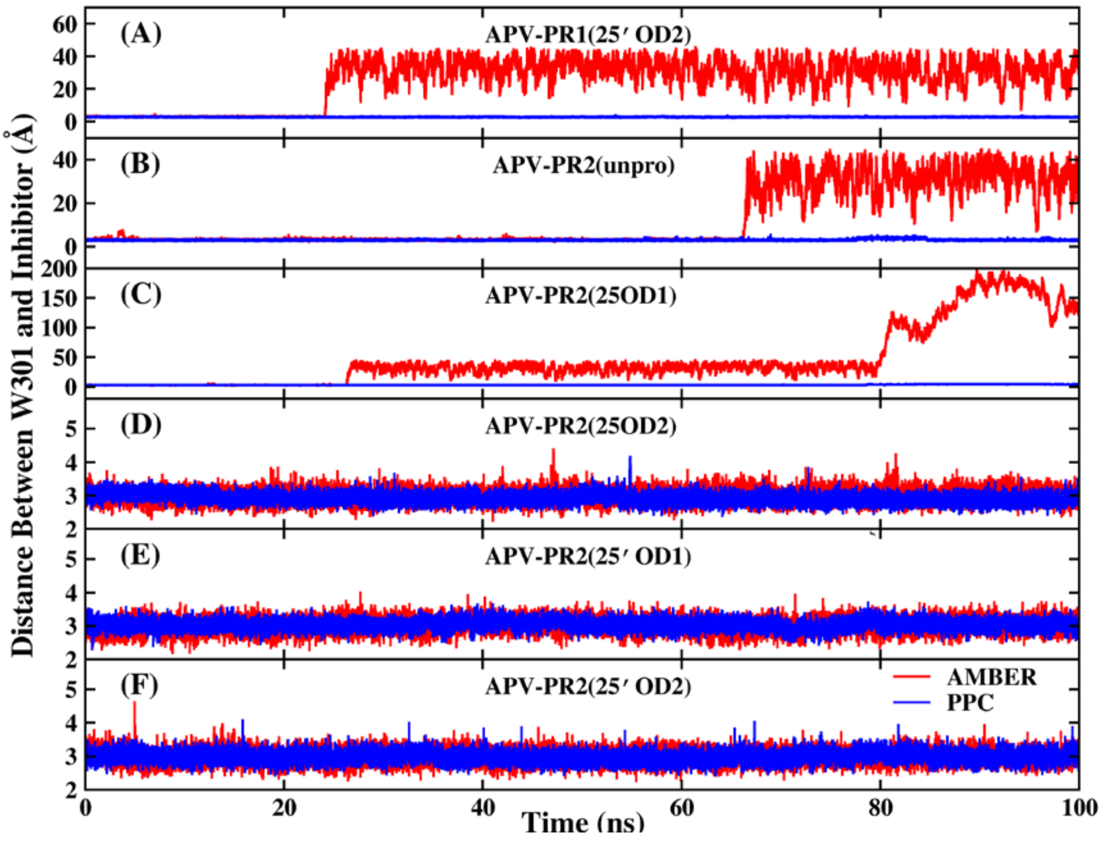

Figure S4
